# The monocyte-to-lymphocyte ratio: Sex-specific differences in the tuberculosis disease spectrum, diagnostic indices and defining normal ranges

**DOI:** 10.1371/journal.pone.0247745

**Published:** 2021-08-30

**Authors:** Thomas S. Buttle, Claire Y. Hummerstone, Thippeswamy Billahalli, Richard J. B. Ward, Korina E. Barnes, Natalie J. Marshall, Viktoria C. Spong, Graham H. Bothamley

**Affiliations:** 1 Department of Respiratory Medicine, Homerton University Hospital, London, United Kingdom; 2 Microbiology Department, Homerton University Hospital, London, United Kingdom; 3 Department of Immunobiology, Blizard Institute, Barts and The London School of Medicine and Dentistry, Queen Mary University of London, London, United Kingdom; 4 Department of Infectious and Tropical Diseases, London School of Hygiene and Tropical Medicine, London, United Kingdom; Rutgers Biomedical and Health Sciences, UNITED STATES

## Abstract

**Background:**

The monocyte-to-lymphocyte ratio (MLR) has been advocated as a biomarker in tuberculosis. Our objective was to evaluate its clinical value and associations.

**Methods:**

Blood counts, inflammatory markers and clinical parameters were measured in patients with and those screened for tuberculosis. Complete blood counts (CBCs) from a multi-ethnic population aged 16 to 65 years were evaluated; a sub-group with normal hematological indices was used to define the range of MLRs.

**Results:**

Multivariate analysis in proven tuberculosis (n = 264) indicated MLR associated with low serum albumin, high white cell counts and a positive culture; values were higher in sputum smear-positive pulmonary tuberculosis (S+PTB). Analysis in S+PTB (n = 296) showed higher MLRs in males and those with high neutrophil counts, low serum albumin and high C-reactive protein. The diagnostic value of MLRs was assessed by comparing notified patients with TB (n = 264) with denotified cases (n = 50), active case-finding in non-contacts (TB n = 111 and LTBI n = 373) and contacts of S+PTB (n = 149) with S+PTB found at screening (n = 75). Sensitivities and specificities ranged from 58.0–62.5% and 50.0–70.0% respectively for optimal cut-off values, defined by ROC curves. In CBCs obtained over one month, ratios correlated with neutrophil counts (ρ = 0.48, P<0.00001, n = 14,573; MLR = 0.45 at 8–8.9 x 10^9^/L) and were higher in males than females (P<0.0001). The MLR range (mean ± 2SD) in those with normal hematological indices (n = 3921: females 0.122–0.474; males 0.136–0.505) paralleled LTBI MLRs. Ratios did not predict death (n = 29) nor response to treatment (n = 178 S+PTB with follow-up CBCs). Ratios were higher in males than female in the 16–45 years age group, where immune differences due to sex hormones are likely greatest.

**Conclusions:**

Severe tuberculosis and male sex associated with high MLRs; the same variables likely affect the performance of other biomarkers. The ratio performed poorly as a clinical aid.

## Introduction

Tuberculosis (TB) was declared a global health emergency by the World Health Organization in April 1993 and remains a leading cause of death and disease [1]. The microbiological diagnosis of TB has advanced considerably through molecular testing [2]. The use of two proteins found in *Mycobacterium tuberculosis* but not in Bacille Calmette-Guérin (BCG) in interferon-gamma release assays (IGRAs) has improved the specificity of diagnosing latent tuberculosis infection (LTBI) [3].

Many non-specific biomarkers have been proposed to identify those with a positive IGRA who will develop active TB [4], those with sub-clinical disease (i.e. those without symptoms often with a normal chest x-ray without raised inflammatory markers) [5], those likely to die from TB [6], those whose genotype might suggest a protective response to *Mycobacterium tuberculosis* [7] and transcriptomic measures of risk, diagnosis and treatment response [8]. The monocyte-to-lymphocyte ratio (MLR) has experienced a revived interest as a biomarker. Monocytes are released from the bone marrow into the bloodstream in sepsis and other infections and migrate to foci of infection where they mature into macrophages [9]. Lymphocytes are responsible for the cellular and antibody responses to infection [10]; for instance, the low number of CD4+ T lymphocytes in HIV infection are responsible for the susceptibility to infections. Originally identified in TB patients, a rabbit model suggested that both high and low MLRs might be markers for TB progression [11]. In a mouse model, high MLRs were associated with impaired protection of BCG against TB [12]. An elevated MLR was thought to correlate with different stages of TB disease [13], identify those with HIV infection most likely to develop active TB [14, 15], contacts likely to develop active TB [16] and neonates at risk of TB [17]. *In vitro* studies with a mycobacterial growth inhibition assay suggested that monocytes and lymphocytes from those with a higher MLR were less able to inhibit the growth of BCG [18]. We, therefore, examined the MLR in patients with TB and LTBI in order to test the hypothesis that such a simple measurement from a complete blood count (CBC) could be of clinical value. Early results were reported in the form of abstracts [19, 20].

## Methods

### Study design

A retrospective audit of the value of MLRs in patients with or screened for tuberculosis. Research was conducted according to the principles expressed in the Declaration of Helsinki. TB patients had given informed written consent in the clinical trial Blood Tests in Tuberculosis III, approved by the East London and City Health Authority Research Ethics Committee (P/03/285), registered under the number NIHR 4177. Those screened for TB had given informed written consent in the UK PREDICT TB Study, whose procedures and protocol were approved by the Brent NHS Research Ethics Committee (10/H7017/14). Both gave access to their clinical data and investigations which were used in this study. For those who were screened for participation in these studies or had provided these data taken during normal clinical care but had not been entered into these trials, waivers were given according to the processes set by the UK Research Institute/Medical Research Council/NHS Health Research Authority (http://www.hra-decisiontools.org.uk/ethics/). In addition, for non-participants of the trials, the study was confirmed as an audit and related to quality improvement of clinical care by the local Research and Development Office.

### Setting

A university district general hospital in an inner-city London borough with a high incidence (falling from 62 to 22 per 100,000 during the period of study) of TB in a multi-ethnic population.

### Participants

**Fig 1:**
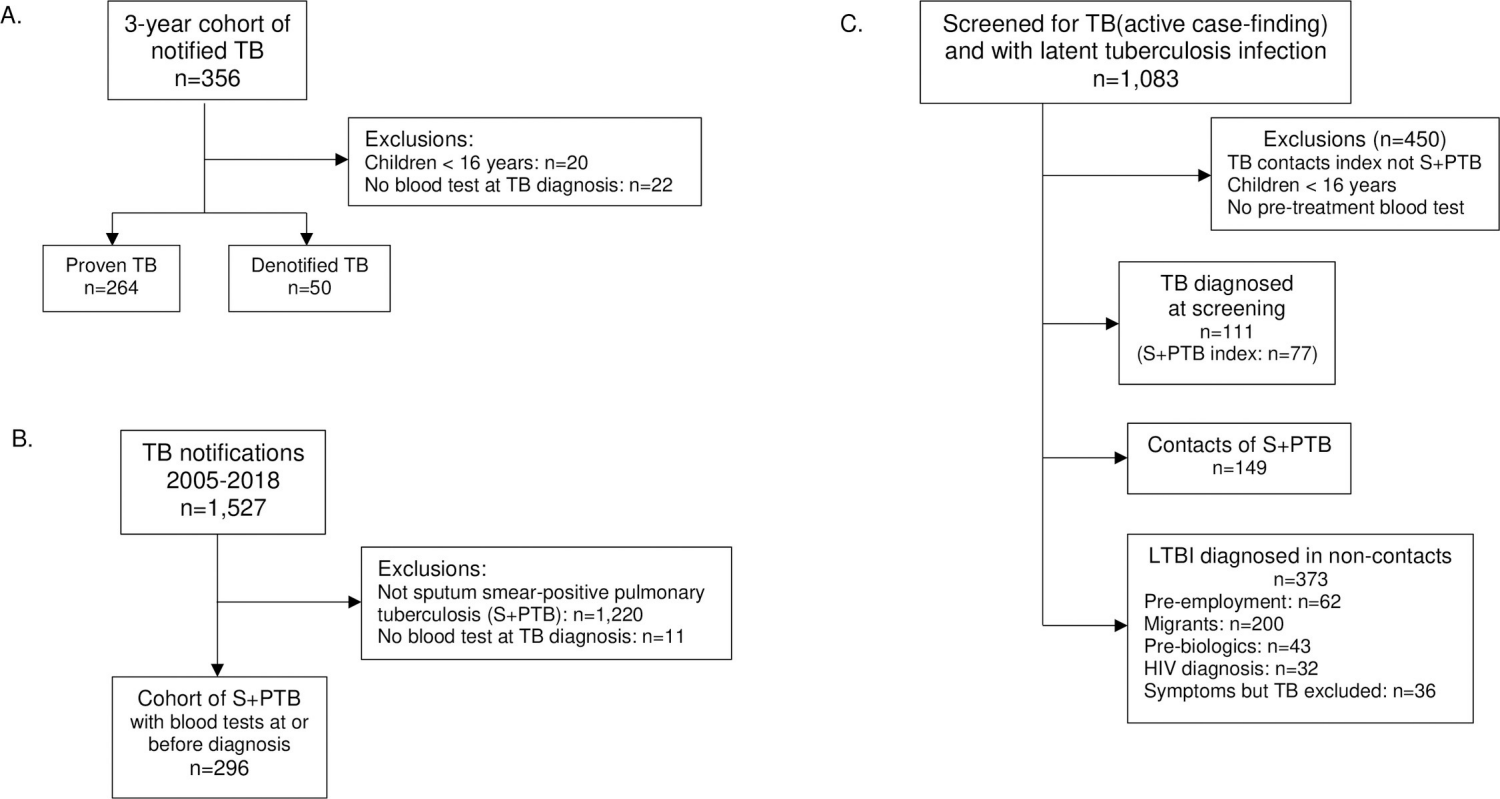
Study populations. Additional demographic details are given in Table 1 and S1 Table in S2 File. A. The cohort of all consecutive TB patients over a 3-year period. B. Selection of a consecutive cohort of patients with sputum smear-positive pulmonary tuberculosis (S+PTB) from all TB patients 2005–2018. C. Active case-finding and description of those with evidence of active or latent tuberculosis infection (LTBI) who were offered either full or preventive treatment respectively.

Patients were eligible if they attended TB Clinics between 2005 and 2018 and had a complete blood count (CBC) before the start of any treatment (Fig 1). Children under 16 years of age were excluded. Active TB was defined by a) a positive culture or by b) a combination of consistent radiology, histology and other tests sufficient for the clinician to notify the patient as having TB and to start a course of treatment without a change in this opinion during treatment nor within the following three years. Contacts were defined by their index case disease type, had spent > 8 h in close proximity to the index case, had attended the TB screening, had a positive IGRA or tuberculin skin test (TST) and were referred for consideration of preventive treatment. LTBI (latent tuberculosis infection) was defined in the screened populations as having a positive IGRA (QuantiFERON > 0.35 IU/mL or positive T-SPOT.*TB*) or a TST ≥ 15 mm for those with a BCG scar and ≥ 10 mm for those without a BCG scar, and without evidence of active TB. All participants constituted consecutive series (Fig 1).

Control groups included those originally notified as having TB who subsequently proved to have another diagnosis (denotified TB), those with LTBI and a anonymized CBCs analyzed during one month in 2018, in whom those with TB or over 65 years on the grounds of likely co-morbidities were excluded. Only data where the hemoglobin, total red cell count, packed cell volume, mean cell volume, mean cell hemoglobin, platelet count, total white cell count, neutrophils, eosinophils, and basophils were all within normal ranges were used (S2 Table in S2 File).

### Variables

TB notification data (age, sex (self-identified), ethnicity (self-identified), country of birth, previous TB, BCG scar, HIV status, diabetes, problem alcohol use, site of disease (patient or index)) were recorded as required by Public Health England for TB as a notifiable disease. Chest radiograph zones out of six and cavitation, sputum smear, days to culture, drug susceptibility test (DST) results, sputum culture at 2 months and end of treatment, and outcome were noted. Deaths were classified as being a) due to TB, i.e. TB was the main cause of death when any other illness at the time was either not present or merely a co-morbidity, such as diabetes; b) TB contributed, i.e. where a clear main cause of death (e.g. pulmonary embolus) was not due to TB, but where TB may have contributed (e.g. rifampicin treatment having induced a hypercoagulable state); or c) clearly unrelated to TB (e.g. a road traffic accident). At screening, reason for attendance, TST and IGRA results were recorded. Blood samples were taken before the start of any treatment, at 2 months and the end of treatment.

### Statistical analysis

Data were analyzed with GraphPad Prism version 7 and Microsoft Excel. Univariate analyses were performed with Student’s t-test for continuous predictors and linear regression with categorical predictors. A cut-off of P < 0.1 was employed to define those predictors to be entered into multiple regression analysis (https://stats.blue/Stats_Suite/multiple_linear_regression_calculator.html). Initial examination included all such predictors, which were then removed individually and added back until the best fit model was determined where all coefficients (excluding those for the constant) were significantly greater than zero.

Receiver operating characteristic (ROC) analysis addressed the optimal cut-off MLR and area under the curve (AUC) values (John Hopkins web-based calculator for ROC curves, http://www.rad.jhmi.edu/jeng/javarad/roc/JROCFITi.html [21]). Cut-off MLRs above the 95^th^ and below the 5^th^ centile of the LTBI and the selected anonymized control groups defined diagnostic indices. Chi-squared analysis assessed the significance of MLRs related to these cut-offs. A power calculation showed that to detect a 10% difference between the sensitivities of the MLR with an 80% power at the 5% level required 199 patients in each group if the higher sensitivity were 20%, or 293 if 30%, but >3000 would be required if the differences between males and females were 5% and the sensitivities lay between 30 and 70% [22].

## Results

### TB patients

356 patients were notified as having TB over the study period. Twenty were children and 13 had no pre-treatment blood test; 50 were subsequently denotified (Fig 1). The firm alternative diagnoses included cancer (5), pneumonia (2), abscesses (3), Crohn’s disease, glomerulonephritis, lupus and autoimmune encephalitis, non-tuberculous mycobacteria, self-healed tuberculosis and LTBI. Demographic details of the remaining 264 patients are given in Table 1.

**Table 1:** Demographic data.

	Active TB	Sputum smear-positive TB	Active TB: diagnosis at screening	IGRA+/TST+ contacts of S+PTB	Other IGRA+ screened	Control group & one month’s clinics
	3-yr cohort					
	(n = 264)					
				(n = 149)	(n = 373)	(n = 3921)
		(n = 296)	(n = 111)			
	n (%)	n (%)	n (%)	n (%)	n (%)	n (%)
**Age**:						
median (range), years	38 (16–88)	36 (16–87)	31 (16–85)	28 (16–67)	35 (16–73)	38 (16–65)
**Female sex**:	88 (33)	101 (34)	55 (50)	72 (48)	196 (53)	2519 (64)
**Ethnicity**:						
Indian subcontinent	80 (30)	41 (14)	25 (23)	26 (18)	59 (16)	NA
Black African	72 (27)	74 (25)	29 (26)	44 (30)	196 (53)	
Afro-Caribbean	28 (11)	52 (18)	20 (18)	14 (14)	14 (4)	
Turkish/Kurdish	23 (9)	18 (6)	1 (1)	2 (2)	32 (9)	
White EU	18 (7)	25 (9)	2 (2)	8 (8)	16 (4)	
White UK	19 (7)	41 (14)	10 (9)	10 10)	19 (5)	
Mixed	4 (2)	11 (4)	8 (7)	1 (1)	1 (0)	
Other	20 (8)	34 (12)	16 (15)	20 (20)	36 (10)	
**UK-born**:	58 (22)	96 (33)	34 (31)	30 (30)	30 (8)	NA
**Previous TB**:	16 (6)	34 (12)	0 (0)	0 (0)	0 (0)	1 (0.03)
**IGRA**:						(Clinics n = 93)*
Positive	137 (84)*	65 (83)*	49 (85)	146 (98)	300 (95)	25 (32)*
Negative	24 (15)*	8 (10)*	8 (14)	1 (1)	0 (0)	41 (53)*
Indeterminate	2 (1)*	5 (7)*	1 (1)	0 (0)	0 (0)	4 (5.1)*
Not done	101 (38)	218 (74)	53 (48)	2 (1)	0 (0)	15 (16)
**BCG**:	195 (79)*	208 (74)*	79 (75)*	NA	NA	NA
**HIV coinfection**:	9 (4)*	20 (7)*	3 (4)*	3 (2)	1 (1)	NA
**Diabetes**:	35 (13)	31 (10)	7 (6)	NA	NA	NA
**Alcohol problem:**	36 (14)	71 (24)	14 (13)	NA	NA	NA
**Site of disease**:						
Pulmonary	91 (35)	296 (100)	62 (56)	1(1)	1(1)	3 (0.08)
Extra-pulmonary	154 (58)	0 (0)	48 (44)	2 (1)	0 (0)	4 (0.10)
Both	19 (7)		1 (1)	0(0)	0 (0)	0 (0)
**Drug-resistance:**						TB clinics
Isoniazid	21 (14)*	61 (21)	8 (14)*			0 (0)
Rifampicin	3 (2)*	6 (2)	1 (2)*	NA	NA	0 (0)
Pyrazinamide	3 (2)*	2 (1)	0 (0)*			0 (0)
MDR-TB	1 (1)*	2 (1)	0 (0)*			0 (0)
**White blood count**						
**> 11 x 10**^**9**^**/L**:	30 (11)	51 (17)	7 (6)	3 (2)	9 (3)	0 (0)

*indicates percentage of those tested or examined or with available data

TB = tuberculosis; IGRA = interferon-gamma release assay; TST = Tuberculin skin test; NA = not available; UK = United Kingdom; BCG = Bacille Calmette-Guérin; HIV = human immunodeficiency virus; MDR-TB = multidrug-resistant tuberculosis

**Fig 2:**
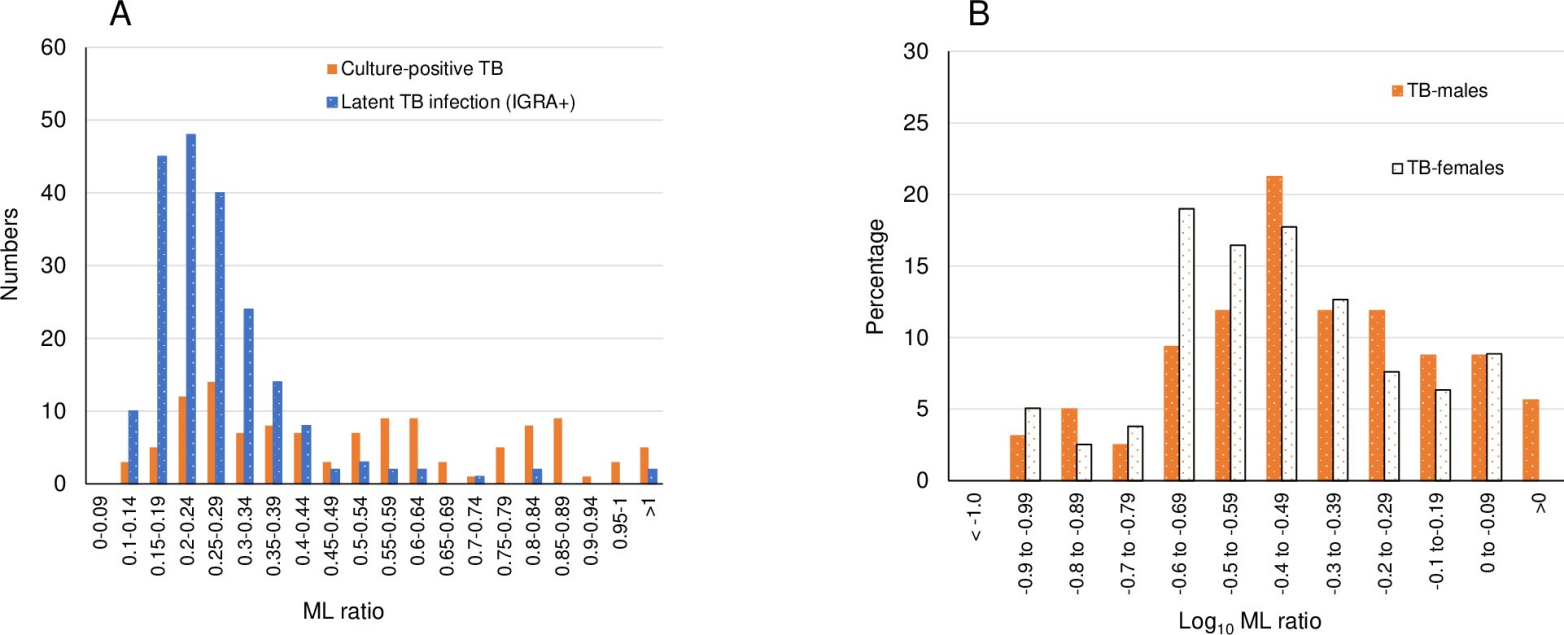
Monocyte-to-lymphocyte ratios (MLRs) and spectrum of tuberculosis (3-year cohort). A. MLRs in patients with culture-positive tuberculosis and screened individuals with a positive interferon-gamma release assay but no active tuberculosis. This shows a right-sided skew of data. B. Log-transformed MLRs in patients with tuberculosis related to sex.

MLRs showed a right-sided skew which was improved by log transformation (Fig 2). Those with culture-positive tuberculosis showed more values in the higher ranges than either those who had other forms of TB or had LTBI.

Clinical variables were examined for any significant association with the MLR (Table 2). Interactions were examined and significant associations were found for smear and culture (coefficient 0.36 ± 0.12, p = 0.008). The best fit model was with albumin, white cell count and culture (Table 2 and S1 File). Addition of log-transformed CRP showed a significant interaction with albumin and review of the CRP data indicated many had a CRP of <5 mg/L, which could not be log. transformed.

**Table 2:** Univariate and multivariable regression analysis of MLRs in tuberculosis.

			Univariate analysis		Multiple linear regression^a^	
		n	MLR	P-value	Coefficient	P-value
			Log mean ± SE		± SE	
Sex	Male	176	-0.38 ± 0.020	0.1130		
	Female	88	-0.43 ± 0.025			
Smear	Positive	67	-0.25 ± 0.034	0.0001	-0.076 ± 0.13	0.555
	Negative	197	-0.44 ± 0.017			
Culture	Positive	157	-0.33 ± 0.021	<0.0001	0.042 ± 0.038	0.268
	Negative	107	-0.49 ± 0.022		(0.095 ± 0.029)^b^	(0.0011)
Site of TB	Pulmonary	103	-0.32 ± 0.028	0.0002	-0.06 ± 0.059	0.345
	Extra-pulmonary	161	-0.44 ± 0.019			
HIV	Positive	9	-0.48 ± 0.104	0.3442		
	Negative	242	-0.39 ± 0.017			
Diabetes	Positive	35	-0.39 ± 0.043	0.8813		
	Negative	229	-0.39 ± 0.017			
Alcohol problem	Yes	36	-0.38 ± 0.047	0.5369		
	No	228	-0.40 ± 0.017			
Drug resistance	Yes	28	-0.41 ± -0.059	0.2030		
	No	130	-0.34 ± 0.020			
BCG scar	Yes	195	-0.39 ± 0.019	0.8778		
	No	54	-0.40 ± 0.032			
	Linear regression analysis					
			R-squared			
Age (years)		263^c^	0.0074	0.1644		
White blood count		264	0.0775	<0.0001	0.013 ± 0.005	0.011
					(0.016 ± 0.005)	(0.0013)
Albumin (g/L)		254	0.257	<0.0001	0.092 ± 0.133	0.49
					(-0.015 ± 0.0021)	(<0.00001)
Globulin (g/L)		251	0.0414	0.001187	-0.011 ± 0.014	0.407
Albumin-to-globulin ratio		250	0.1758	<0.0001	-0.018 ± 0.493	0.971
Log CRP (mg/L)		174	0.2709	<0.0001	-0.335 ± 0.274	0.223

^a^ Data are given for the initial model with the

^b^ best-fit model in brackets

^c^ One outlier with a monocyte count of 8 was excluded

In view of the significant difference in proportion of males and females with TB, the sexes were also analyzed separately. An improved model was found for males using predictors of albumin, culture, HIV co-infection and albumin-to-globulin (AG) ratio, the latter two predictors replacing white cell count (adjusted R-squared 0.395 compared to 0.328; total sum of squares 11.533 compared to 11.974; F = 26.478 compared to 28.003; S1 File). The negative coefficient for HIV in males was associated with low monocyte counts (range 0.1 to 0.5). Surprisingly, the AG ratio did not show a significant interaction with albumin nor with globulin and its coefficient of association with log MLR was positive. No additional improvement above albumin, culture and white cell count could be found for females, although for this group there was a significant interaction between culture and albumin values in females (P = 0.026; S1 File).

### MLRs in smear-positive pulmonary tuberculosis (S+PTB)

**Fig 3:**
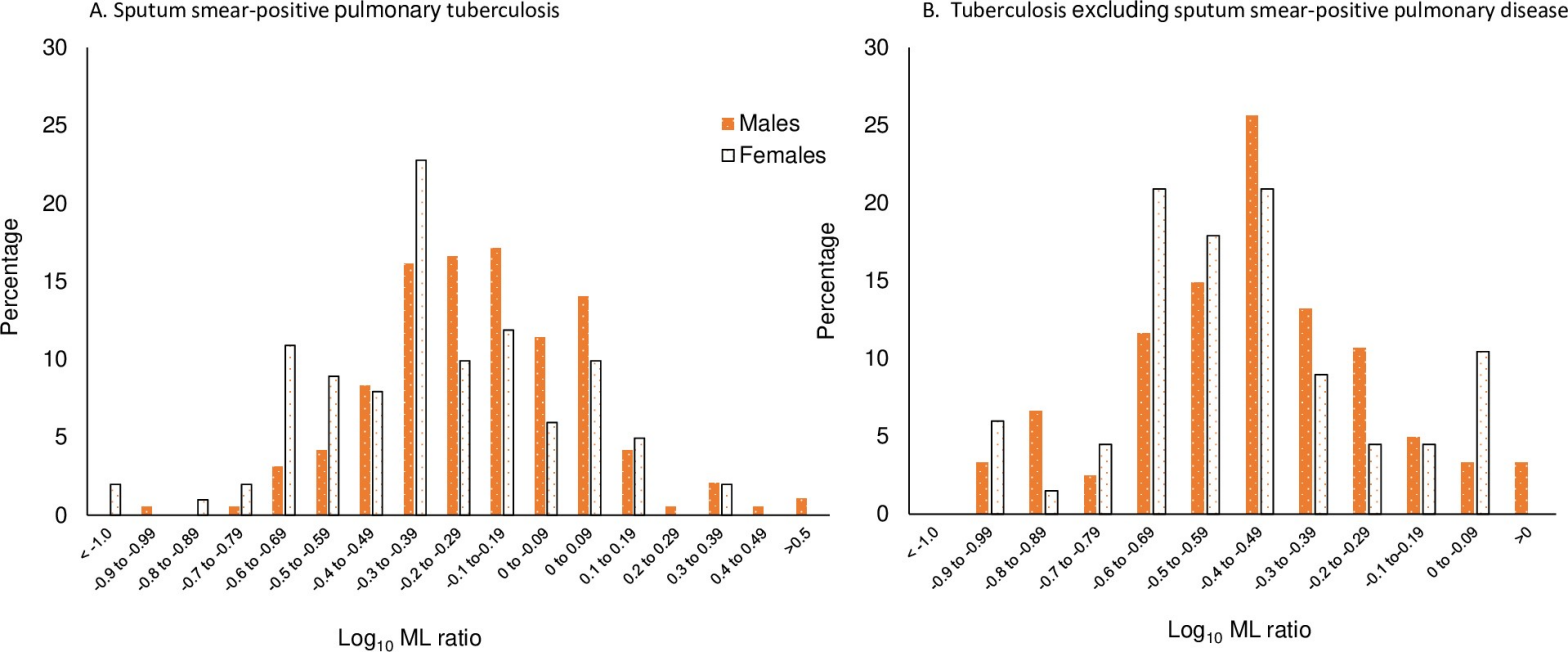
Monocyte-to-lymphocyte ratios and sex. A. Patients with sputum smear-positive pulmonary tuberculosis. B. Patients with tuberculosis excluding sputum smear-positive pulmonary disease. Males show higher values than females.

Patients with S+PTB had shown the highest MLRs (Table 2). We therefore wished to explore whether the preliminary findings in the tuberculosis cohort could be refined by further examining this group (Table 3). In particular, we wanted to know whether the association with the white cell count pertained mainly to the neutrophil count and whether the time to culture, an indicator of bacterial load, was a significant predictor for the MLR. This group would also permit further exploration of other variables that might be associated with higher MLRs, considering the lesser overlap of values from those with LTBI compared to other patients within the TB spectrum. Demographic details of the make-up of this group are given in Table 1 and S1 Table in S2 File. We also expected that this group might shed more light as to why higher MLRs are found in males than females (Fig 3).

**Table 3:** Univariate and multivariable regression analysis of MLRs in S+PTB.

			Univariate analysis		Multiple linear regression^a^	
		n	MLR	P-value	Coefficient	P-value
			Log mean ± SE		± SE	
Sex	M	193	-0.19 ± 0.018	0.0005	0.11 ± 0.034	0.001
	F	100	-0.30 ± 0.028		(0.1 ± 0.029)	(0.001)
Cavities	Present	174	-0.20 ± 0.018	0.0827	0.10 ± 0.097	0.307
	Absent	117	-0.26 ± 0.027			
HIV	Positive	18	-0.23 ± 0.078	0.9360		
	Negative	231	-0.22 ± 0.017			
Diabetes	Positive	31	-0.24 ± 0.037	0.7332		
	Negative	262	-0.23 ± 0.017			
Alcohol problem	Yes	74	-0.22 ± 0.035	0.7648		
	No	219	-0.23 ± 0.017			
Drug resistance	Yes	66	-0.23 ± 0.036	0.7504		
	No	221	-0.22 ± 0.017			
BCG scar	Yes	206	-0.23 ± 0.018	0.6696		
	No	74	-0.22 ± 0.030			
	Linear regression analysis					
			R-squared			
Smear grade (df = 4)		274	0.0123	0.0668	-0.06 ± 0.037	0.585
Log time to culture (d)		283	0.0026	0.3927		
CXR (up to 6 zones)		281	0.0125	0.0613	-0.04 ± 0.034	0.223
Age (years)		293	0.0019	0.4572		
White blood count		293	0.0224	0.0103	Not done	
Neutrophil count		293	0.0522	0.00009	0.02 ± 0.005	0.003
					(0.02 ± 0.005	(0.001)
Albumin (g/L)		288	0.1283	< 0.00001	-0.02 ± 0.017	0.408
					(-0.12 ± 0.03)	(0)
Globulin (g/L)		286	0.0476	0.0002	0.01 ± 0.015	0.389
Albumin-to-globulin ratio		286	0.1244	< 0.00001	0.49 ± 0.64	0.443
Log CRP (mg/L)		249	0.1327	< 0.00001	0.03 ± 0.35	0.934
					(-0.41 ± 0.18)	(0.02)

^a^ Data are given for the initial model. The best fit model figures are included in brackets, initially including log CRP (S1 File). However, due to the greater number of missing values, re-analysis was performed without log CRP for other predictors and again analyzing for a best fit model.

Serum albumin remained an important negative predictor of the MLR. The association with neutrophil count was greater than with the white cell count. However, the time to culture was not a predictor. The best fit model for log MLRs included the predictors of sex, albumin, log neutrophil count and log CRP, although there was a significant interaction between albumin and log CRP (P = 0.001; S1 File).

Regarding the difference between the sexes, univariate analysis for the different variables is given in Table 4. Notably, variables which show a significant association in females were often not significant in males. This accords with Fig 3A, where the departure from a normal distribution of values is more apparent in the female S+PTB population. Multiple linear regression analysis for females indicated a best fit model for predictors of log. neutrophil count, albumin-to-globulin ratio, smear and cavities, whereas for males only log. CRP and albumin were valuable and interacted significantly with each other (P = 0.026; S1 File).

**Table 4:** Univariate analysis of variables by sex in S+PTB.

			Females			Males	
		n	MLR	P-value	n	MLR	P-value
			Log mean ± SE			Log mean ± SE	
Cavities	Present	53	-0.25 ± 0.046	0.0495	122	-0.20 ± 0.022	0.6066
	Absent	46	-0.38 ± 0.044		71	-0.18 ± 0.031	
HIV	Positive	8	-0.63 ± 0.21	0.0063	11	-0.10 ± 0.10	0.2457
	Negative	78	-0.29 ± 0.032		153	-0.19 ± 0.019	
Diabetes	Positive	13	-0.26 ± 0.078	0.453	18	-0.23 ± 0.033	0.4230
	Negative	88	-0.33 ± 0.035		175	-0.19 ± 0.019	
Alcohol problem	Yes	17	-0.45 ± 0.12	0.067	58	-0.18 ± 0.037	0.8321
	No	84	-0.29 ± 0.029		135	-0.19 ± 0.020	
BCG	Yes	63	-0.34 ± 0.042	0.483	143	-0.20 ± 0.021	0.3590
	No	33	-0.30 ± 0.048		40	-0.16 ± 0.036	
Drug resistance	Yes	25	-0.43 ± 0.090	0.026	42	-0.15 ± 0.15	0.3388
	No	74	-0.27 ± 0.030		147	-0.20 ± 0.021	
Linear regression analysis							
			R-squared			R-squared	
Smear grade (df = 4)		94	0.0818	0.0052	181	0.0004	0.7893
Log time to culture (d)		97	0.0265	0.1111	186	0.0021	0.5348
CXR (up to 6 zones)		96	0.0166	0.2107	185	0.0107	0.1609
Age (years)		100	0.0006	0.8088	193	0.0059	0.2884
White blood count		100	0.0735	0.0064	193	0.0031	0.4417
Neutrophil count		100	0.1232	0.0028	193	0.0183	0.0627
Log neutrophil count		100	0.1411	0.0001	193	0.0065	0.2652
Albumin (g/L)		98	0.1226	0.0004	190	0.1331	<0.0001
Globulin (g/L)		97	0.0439	0.0395	189	0.0438	0.0039
AG ratio		97	0.1739	0.0002	189	0.1055	<0.0001
Log CRP (mg/L)		79	0.2099	0.0002	169	0.1124	<0.0001

### Diagnostic indices

**Fig 4:**
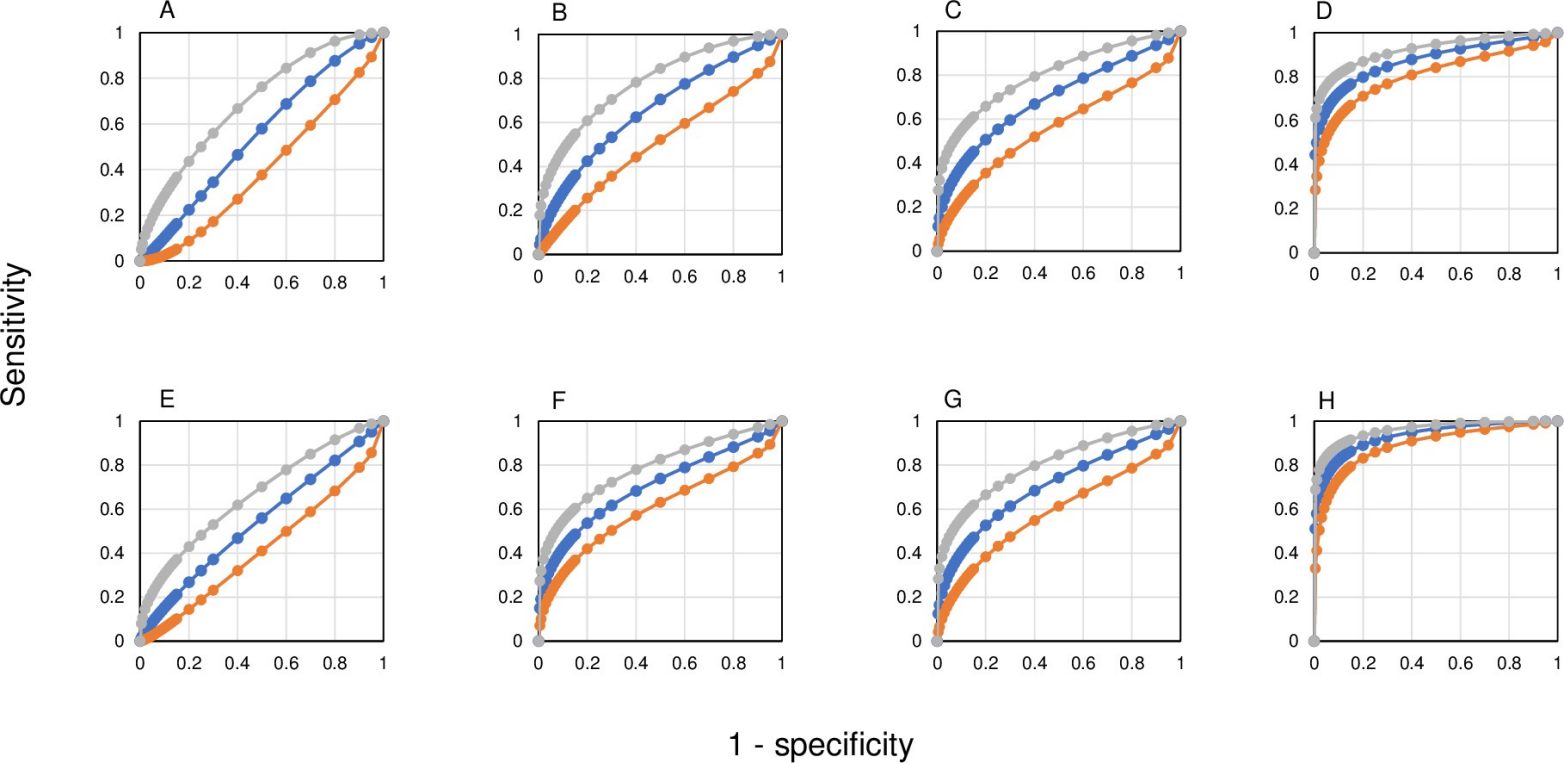
Receiver-operating characteristics for MLRs by sex and patient group. Females are represented in the upper line (**A**, **B**, **C and D**) and males in the lower line (**E**, **F**, **G** and **H**). **A** and **E** compare notified cases, TB and denotified. **B** and **F** compare TB found on screening (active case-finding) with screened LTBI, excluding contacts. **C** and **G** compare S+PTB found at screening with LTBI in contacts of S+PTB cases. **D** and **H** compare all S+PTB (active and passive case-finding) with LTBI in contacts of S+PTB cases. See Table 5 for values of the area under the curve (AUC) and optimal diagnostic indices.

Screened persons with LTBI were identified from records of those offered preventive treatment (Fig 1). Contacts of patients with S+PTB (n = 149, 146 without TB) were considered separately (Table 1) as being most likely to develop active disease as a result of recent infection. For more distant infection, only screened individuals with a positive IGRA were included (n = 373, 372 without TB). The MLR for those with LTBI, whether with recent or distant exposure, showed an almost normal distribution with data skewed towards higher ML values (Fig 2A). Log-transformation of the data showed a normal distribution. Assessing receiver-operating characteristics (ROC), the areas under the curve (AUC) varied according to the comparison made (Fig 4). The shortest distance of the curve to the top left-hand corner was used to define the optimal diagnostic indices (Table 5).

**Table 5:** Definition of best diagnostic points from ROC curves.

Population	Sex	TB	Not TB	AUC ± SE	Best diagnostic point		MLR cut-off value
		(n)			Sensitivity	Specificity	
			(n)		(%)	(%)	
Notified initially as TB	F	88	23	0.55 ± 0.067	58.0	50.0	0.316
	M	176	27	0.55 ± 0.058	56.0	50.0	0.381
All screened excluding contacts	F	20	193	0.66 ± 0.069	62.5	60.0	0.289
	M	56	176	0.70 ± 0.043	61.7	70.0	0.375
Contacts S+PTB vs S+PTB found at screening ^b^	F	36	70	0.69 ± 0.056	59.6	70.0	0.333
	M	44	74	0.71 ± 0.051	61.3	70.0	0.381
Contacts of S+PTB vs S+PTB	F	102	70	0.88 ± 0.026^a^	76.7	85.0	0.333
	M	195	74	0.93 ± 0.015^a^	84.1	88.0	0.375

^a^ Significant difference, P = 0.0266

^b^ Active case-finding only

However, the positive likelihood MLRs in whatever category remained <10, indicating that the MLR would not be a valuable predictor for TB in common clinical situations.

### Could higher cut-off values of the MLR be useful?

One of the problems in evaluating the literature on the MLRs is to know whether the values that are given might be expected in a “normal” population or do indeed reflect the pathology of TB. Fig 2 indicates a considerable overlap in MLRs between LTBI and active TB, with higher MLRs in those with TB. Comparing the screened LTBI population MLRs, there was no significant difference between contacts and other screening (P-values > 0.4). These two populations of LTBI were therefore combined to develop an upper limit for MLR defined as the log. mean ± 2SD, giving estimated cut-off values of 0.483 (95% CI 0.462 to 0.506) for females and 0.504 (95% CI 0.461 to 0.551) for males.

In order to check the validity of the cut-off values, we examined all anonymized CBCs taken during a single month from those aged 16–65 years (n = 14,573). The MLR increased according to the neutrophil count (ρ = 0.48, P<0.00001); those with an MLR of 0.45 corresponded to those with a neutrophil count of 8–8.9 x 10^9^/L, the laboratory upper limit of normal being 8 x 10^9^/L (S1 Fig). The MLR showed a distribution with a right-sided skew, which was partly normalized by log transformation. A box plot indicated that there still remained a skew toward higher values. Plotting histograms for monocytes and lymphocytes indicated that the former showed an almost normal distribution, but higher monocyte counts were responsible for most of the skewing. A QQ plot confirmed this impression (S2A Fig). Indeed, high monocyte values have been associated with a number of clinical conditions [23, 24]. On the assumption that other hematological indices might be affected in disease, all samples with any value outside the normal range except for monocytes and lymphocytes were first removed, leaving 4,254 CBCs. If samples were selected with normal hematological indices apart from the monocyte and lymphocyte counts, then upper limit would have been 0.515 (95% CI 0.511 to 0.518) for females and 0.595 (95% CI 0.584 to 0.607) for males and few TB patients had positive values (S3 Table in S2 File). Moreover, the MLR values were significantly greater than those derived from the LTBI population (P = 0.0003). A QQ plot indicated significant departures from normality, which was especially significant in males (S2B and S2F Fig). Excluding CBCs to those with a lymphocyte count > 4 x 10^9^/L and monocytes to > 1 x 10^9^/L still showed skewing on a QQ plot and a significant difference from the LTBI population. A monocyte count > 0.8 x 10^9^/L has been associated with significant clinical disease in an emergency room [24]. Using a lymphocyte count > 4 x 10^9^/L and monocytes to > 0.8 x 10^9^/L defined a population close to a normal distribution as well as with the closest approximation to the LTBI population and this was therefore used to define the cut-off limits (S2D, S2G and S3 Figs). CBCs from the TB clinics were removed from this last population. There remained a significant difference in the log_._ MLRs between the sexes (mean (SD): female -0.619 (0.148); male, -0.582 (0.143), P < 0.0001). Defining cut-off values as log. mean ± 2SD, for females the upper MLR limit was 0.474 (95%CI 0.469 to 0.482) and for males was 0.505 (95% CI 0.497 to 0.515). The sensitivities and specificities derived using these values for the different populations are given in S4 Table in S2 File, but positive likelihood ratios were all < 10.

In patients with all forms of TB within the 3-year cohort, six patients had an MLR below the lower cut-off limits, two with culture-positive TB; two had diabetes, one HIV co-infection (culture-positive), one an alcohol problem, one with malnutrition (culture-positive) and one with no co-morbidities. Using the upper MLR cut-off values, the associations observed in the univariate analysis (Table 2) were confirmed. More patients with culture-positive TB from any site had a high MLR (48/69 (70%) vs. 48/106 (24%); χ^2^ = 10.7, P = 0.001) compared to those without a positive culture. More patients with pulmonary disease had MLRs above the upper limit compared to those with extrapulmonary disease (46/94 compared to 44/159; χ^2^ = 10.8, P = 0.001, see Fig 3). Three patients with miliary TB had MLRs of 0.10 (lymphocytes 5.1 x 10^9^/L), 0.36 and 1.00 (bone marrow depression with lymphocytes and monocytes both with an absolute value of 0.2 x 10^9^/L). Three patients with problem alcohol use and disseminated TB had high MLRs due to lymphopenia (all three had a lymphocyte count of 0.6 x 10^9^/L).

In the group of patients with S+PTB, the MLRs above the cut-off again confirmed the univariate and multiple regression analysis (Table 3): white cell counts >11 x 10^9^/L were more common in those with MLRs above their sex-specific limits (19/96 vs. 10/168; χ^2^ = 12.0, P = 0.0006); albumin levels were more commonly <40 g/L in those with a high MLR (80/90 compared to 103/167; χ^2^ = 18.9, P = 0.00001) and globulin >32 g/L were higher (80/90 compared to 133/163; χ^2^ = 4.9, P = 0.027); C-reactive protein (CRP) levels were high (>10 mg/L) in those with MLRs above the sex-specific limits (74/87 vs. 61/140: χ^2^ = 38.3, P < 0.00001) and higher in males with values >10 mg/L than females (Mann-Whitney, z = 2.007, P = 0.045). However, higher ratios were still more frequent in males (130/194 cf. 56/101; χ^2^ = 3.81, P = 0.05). Those with high MLRs were more likely to have a sputum smear of 3+ (χ^2^ = 9.0, P = 0.0027), but the difference between males and females was not significant. There was no difference in the radiographic extent of disease, but cavitation was more frequent in females with high compared to normal MLRs (χ^2^ = 10.0, P = 0.0016) and in males than females with normal MLRs (χ^2^ = 9.6, P = 0.00195). High or low MLRs were not associated with drug resistance.

During the same month as the anonymized samples, 8 patients with notified tuberculosis had 11 blood tests (Fig 1). In the various TB clinics, 93 individuals were seen, several on more than one occasion: none with LTBI or cured TB had an MLR above the upper limit for their sex, although one patient with sarcoidosis had an MLR below the lower limit. Those with a positive TST or IGRA did not have higher MLRs than those who were negative.

### Prognostic value of the MLR in those with LTBI

In contacts of index cases with S+PTB, there was one patient with an MLR of 1.0 who on review was considered to have active TB, based on atelectasis in left lower zone, TST conversion from 6 to 15 mm induration and a CRP of 28 mg/L and whose chest radiograph cleared following treatment for TB (Table 2). Two other patients, who developed culture-positive TB, had MLRs of 0.26 and 0.22 at screening.

There were 11 contacts of S+PTB who showed a completely negative response to tuberculin at their first visit with a positive TST at 6–8 weeks follow-up. Eight of nine with an IGRA were positive (one rising from 0 to 0.5 IU/mL). All these with evidence of recent infection with TB had an MLR within the normal ranges and none developed TB (all received preventive treatment).

In the IGRA+/TST+ screened population without known contact with TB, 8 had a high MLR (S4 Table in S2 File). One had initially been notified as a case of TB being a contact of an uncle, who had active but not pulmonary TB; this person had several risk factors for TB, minor gastro-intestinal symptoms and was given a trial of standard treatment which was stopped at 2 months (2 months of rifampicin and pyrazinamide being a proven preventive treatment for LTBI). The other 7 did not develop TB, having received preventive treatment.

### Deaths and MLR

There were 29 deaths (1.9%) in notified TB cases from 2005–2017. Three were due to TB, three where TB contributed to death and the remainder were unrelated to TB. MLRs above the sex-specific limits were found in 18 (62%) but were not significantly higher or more numerous in those where TB played a role (5 males and 1 female) compared to those where there was another cause of death. MLRs measured within a day or two of death did not improve on the predictive value above samples taken at the time of TB diagnosis.

### Effect of treatment on MLRs

Two-week follow-up MLRs were available for 178 with S+PTB. Five showed no change, 57 were higher and 115 were lower; MLRs were more likely in males than females to fall to the normal range (χ^2^ = 6.5, P = 0.011). Looking only at results obtained at the start of treatment, at 2 months and at the end of treatment, there was a significant fall in the MLR by 2 months (paired t-test: t = 7.5, P < 0.0001, df 136) and 6 months (t = 5.3, P<0.0001, df 117). Looking at individual patients, a persistently high MLR could not be used to predict who would remain culture-positive at 2 months or have a poor outcome. Four patients had extended inpatient stays and multiple blood tests taken due to their severity of disease. Three showed a reduction in MLR from high to normal values by days 2, 6 and 21 respectively, whilst another showed an MLR which remained high for 247 days. No additional effect was seen in patients with drug-resistant TB.

### Effect of age on MLRs

In order to determine whether the sex difference might be related to sex hormones or differences in inflammatory responses with age, MLRs were divided into age groups 16–45 years (women should be pre-menopausal), 46–65 years and >65 years. In order to ensure that the level of disease burden was comparable, only those with S+PTB were examined. The 16–45 years age group showed the greatest difference in MLR between the sexes (P = 0.0035), although the 46–65 years age group was also significantly different (P = 0.047; Fig 4).

## Discussion

In TB, MLR was associated with a low serum albumin, high white cell count and a positive culture, as well as with high CRP. Males also showed an association with HIV and the serum albumin-to-globulin ratio. In S+PTB, this was refined to a high neutrophil count (females only), with albumin and a high CRP (a significant interaction in males) and male sex. Females with S+PTB also showed a link between MLR and smear and lung cavities. The sensitivity and specificity of MLRs were poor for the diagnosis of TB or as a screening tool to detect active TB in contacts and IGRA+ individuals. High MLRs did not predict the development of TB in screened populations, nor treatment effectiveness, although MLRs usually fell with treatment. MLRs correlated with neutrophil counts in the one month CBC population. High MLRs were more often due to monocytes, especially in males. The sex difference in MLRs was most marked in the 16–45 years age group.

### Sex-specific differences

Males had higher MLRs than females. Males have more S+PTB, whereas the sex ratio in extrapulmonary TB is equal [25, 26]. Lung cavities are more common in males than females [27]. Many of the reported data on MLRs either had a predominance of males in the TB group [13, 28] or did not give sex-specific data [18]. The immune response in males is less effective than in females despite greater TLR2 and TLR4 expression, more Th17 cells and CD4+ Treg cells, CD8+ T cells and NK cells [29]. Monocytosis is a feature of infection [23] and high values are found especially in males [24]. Thus, the higher MLRs in males and their association with more severe forms of TB and with lung cavities might be expected as a sex-specific immune response. Such individuals are more readily diagnosed and regularly bias studies in TB towards a male-predominant population.

### Clinical value

The early use of the MLR in TB noted that “the … ratio cannot … be used as a criterion for diagnosis”, the “monocyte-lymphocyte ratio … is not a sensitive index of activity in the tuberculous patient” (activity being measured by the extent of disease, tachycardia, fever, clinical judgment of prognosis and follow-up over two years) and that “a considerable number of patients with tuberculosis will have a normal monocyte-lymphocyte ratio” [30]. These results from the pre-antibiotic era of TB treatment have been confirmed in a modern setting by this study. The association of lymphopenia and a lower MLR with TB in a rabbit model [11, 31] was rarely observed in our study, even in those with miliary disease as the human comparator of the experimental method.

A low or high MLR has been associated with a hazard ratio of 2.47, 1.5 and 1.22 for the development of TB during follow-up of adults with HIV infection before anti-retroviral therapy [14], in HIV-infected post-partum women [15] and in infants [17] in South Africa, respectively. However, the sensitivities of cut-off values were low (< 10%), such that the MLR had little predictive value for an individual person. A higher hazard ratio (4.5) was observed in contacts who went on to develop TB, but a combination of a tuberculin skin test ≥ 14 mm (sensitivity 7.5%) with monocytes >7.5% of all white blood cells (sensitivity and specificity about 75%) was reported to have a hazard ratio of 8.78, noting that many of the diagnoses of TB were a decision to treat in young children [16]. Using the same criteria from previous studies [16, 28] in our group of contacts, the sensitivities were greater but the specificities were poorer than with our cut-offs and the positive predictive values were lower (S5 and S6 Tables in S2 File).

### Limitations

This audit was performed at a single site. However, this ensured that data collection was uniform and follow-up data consistently available. Few patients had HIV co-infection and those with HIV were taking antiretroviral therapy and most had normal lymphocyte counts (four were < 1 x 10^9^/L). Children were excluded, but this avoided a bias in terms of empirical treatment for TB and a population in which blood tests are fewer and for highly selected reasons. Those over 65 years of age, excluded in the control group, are likely to have disease co-morbidities, especially malignancy, ischemic heart disease and metabolic syndrome [32, 33], which are associated with higher MLRs. Older persons also experience “inflammaging”, having a higher inflammatory response than younger adults [10, 34, 35].

### Generalizability

The use of a real-life group of TB patients and contacts from many ethnic backgrounds encourages extrapolation to the global HIV-uninfected TB population. Analysis of groups by smear and culture status, ensured that both real-life results and data uncontaminated by variations in the diagnosis of “clinical TB” could be obtained.

### Implications

**Fig 5:**
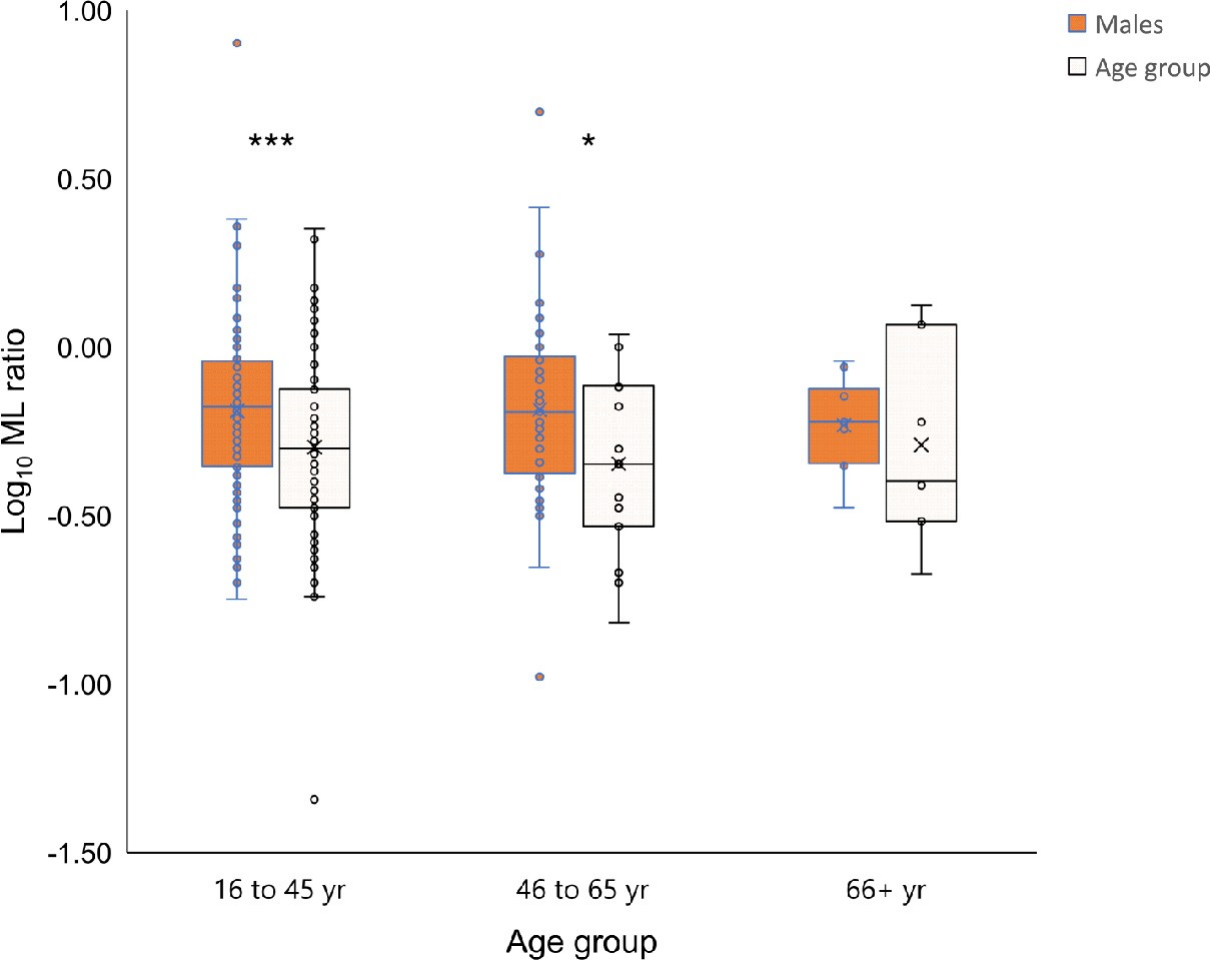
Age and sex differences in monocyte-to-lymphocyte ratios. MLRs were compared using log-transformed values by Student’s t-test. * P < 0.047 and *** P < 0.0035. Mean ML values were 0.65 for males in the 16–45 and 46–65 age groups and 0.50 and 0.45 in females, respectively.

This study has shown the importance of sex as a variable in the evaluation of biomarkers. Such differences touch upon lipid and metabolic differences in cardiovascular disease [36], cancer biomarkers [37] and studies distinguishing general differences between the proteome [38] and transcriptome [39, 40] between the sexes. Both estrogens and androgens affect immune responses [29, 41]. Our data suggested that the difference in MLRs might be due to sex hormones rather than an X-linked cause or sex-related differences in inflammaging due to the differences seen among the age groups, most especially those aged 16–45 years where differences in sex hormones are most apparent (Fig 5). Thus, diseases with a female or male predominance, such as autoimmune diseases or cancer respectively, may need to address the relative efficacy of diagnostic markers and prognostic indicators between the sexes.

Biomarkers and the sexes are especially problematic in tuberculosis. The male predominance is found almost entirely in S+PTB [25, 26] and therefore patient selection will be affected by the number with this form of disease. The relative ease of diagnosis of this form of TB again means that follow-up studies of, say, those with LTBI who go on to develop TB will mean that prognostic biomarkers will be biased towards males.

Interleukin-6 (IL-6) is important in the mobilization of monocytes from the bone marrow [9]. Transcriptomic studies examining the genetics of high MLRs suggested a link with both IL-6 and interferon type I [18]. However, a closer link has been shown with an IL6/IL6R/CEBP gene module, also associated with monocyte expansion, high CRP and a positive sputum smear [42]. These associations are consistent with the findings of the multivariate analyses in this study.

Transcriptomic data investigating the pathogenesis of active TB disease show sex-specific responses [43, 44]. Estrogens, via the alpha receptor, downgrade IL-6 expression in human monocytes [45]. The 3-gene transcriptomic signature of Sweeney, which performed better than 15 other signatures for discriminating latent from active TB [46], has genes whose expression are all affected by sex. Dual-specificity phosphatase 3 (DUSP3) deletion protects female but not male mice from endotoxin shock due to a dominance of M2 (non-classical and anti-inflammatory) macrophages [47]. Guanylate-binding protein 5 (GBP5) is a marker of IFNγ-induced classical monocytes [48], which are more frequent in males, and has been found in those who develop innate immunity after an IFNγ response [49]. Kruppel-like factor 2 (KLF-2), in association with the male-dominant cytokines IL-6 and monocyte chemoattractant protein-1, promotes inflammation in response to tumor necrosis factor (induced by mycobacterial lipoarabinomannan) [50]. If biomarker studies select a majority of males and/or those with S+PTB, their value should be less in females and those with more limited disease.

## Conclusion

The MLR is neither diagnostic nor typical of TB. It shows a bias towards males with S+PTB and other clinical markers of severe disease. These findings might account for its association with S+PTB and its previous popularity as a biomarker in tuberculosis and suggests that other biomarkers may face similar biases.

## Supporting information

S1 Fig Monocyte-to-lymphocyte ratio (MLR) and neutrophil count. Comparison of MLR and neutrophil count in 14,533 CBCs. Error bars were too small to be visible.
(PPTX)

S2 Fig QQ plots for log. ML ratios for unselected and those with normal hematological indices. **A.** All anonymized samples: females n = 9972; males n = 4583. From left to right, QQ plots are separated by sex with females on the top line (**B**, **C**, **D**) and males beneath (**E**, **F**, **G**). **B** and **E** are all anonymized samples; **C** (n = 2661) and **F** (n = 1593) are those with normal hematological indices, but without any limits for monocytes and lymphocytes; **D** and **G** are those with normal hematological indices, but with upper limits of 0.8 x 10^9^/L for monocytes and 4 x 10^9^/L for lymphocytes in females (n = 2550) and males (n = 1426) respectively (TB clinic CBCs not removed).
(TIF)

S3 Fig Comparison between screened population and CBCs: Effect of choosing different limits for the monocyte and lymphocyte counts. **A**. MLRs comparing culture-positive TB, LTBI and hospital CBCs with normal hematological indices. **B**. Log. transformed MLRs: all CBCs. **C**. Log. transformed MLRs: CBCs with normal hematological indices but no restriction on monocyte or lymphocyte limits. **D**. Log. transformed MLRs: CBCs with normal hematological indices, monocytes < 0.8 x 10^9^/L and lymphocyte < 4.0 x 10^9^/L.
(TIF)

S1 File Multivariate analyses of patient predictors and MLR. (DOCX)

S2 File Collated supplemental tables and figure legends. (DOCX)

S3 File Anonymised CBCs. (XLSX)

S4 File Clinical data. (XLSX)

S5 File Data dictionary. (XLSX)
