# Supplementary figures and images for: The monocyte-to-lymphocyte ratio: Sex-specific differences in the tuberculosis disease spectrum, diagnostic indices and defining normal ranges
Source: PLoS One. 2021 Aug 30;16(8):e0247745. doi: 10.1371/journal.pone.0247745 (PMC8405018; doi:10.1371/journal.pone.0247745)

## Slide 1
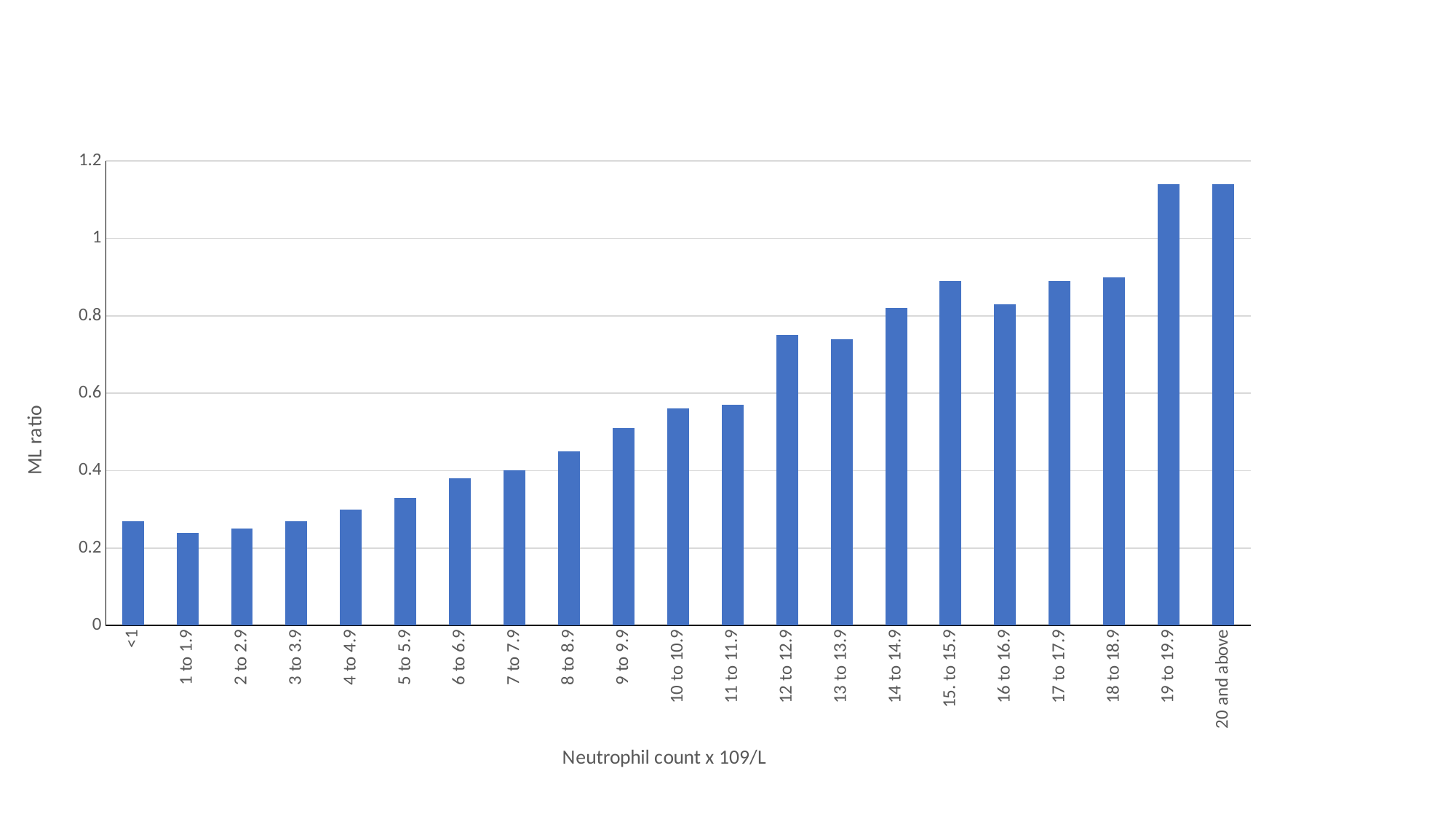

### Chart
| Category | mean ML ratio |
|---|---|
| <1 | 0.27 |
| 1 to 1.9 | 0.24 |
| 2 to 2.9 | 0.25 |
| 3 to 3.9 | 0.27 |
| 4 to 4.9 | 0.3 |
| 5 to 5.9 | 0.33 |
| 6 to 6.9 | 0.38 |
| 7 to 7.9 | 0.4 |
| 8 to 8.9 | 0.45 |
| 9 to 9.9 | 0.51 |
| 10 to 10.9 | 0.56 |
| 11 to 11.9 | 0.57 |
| 12 to 12.9 | 0.75 |
| 13 to 13.9 | 0.74 |
| 14 to 14.9 | 0.82 |
| 15. to 15.9 | 0.89 |
| 16 to 16.9 | 0.83 |
| 17 to 17.9 | 0.89 |
| 18 to 18.9 | 0.9 |
| 19 to 19.9 | 1.14 |
| 20 and above | 1.14 |

Supplement: S1 Fig — Comparison of MLR and neutrophil count in 14,533 CBCs. Error bars were too small to be visible. (PPTX) [file pone.0247745.s001.pptx]

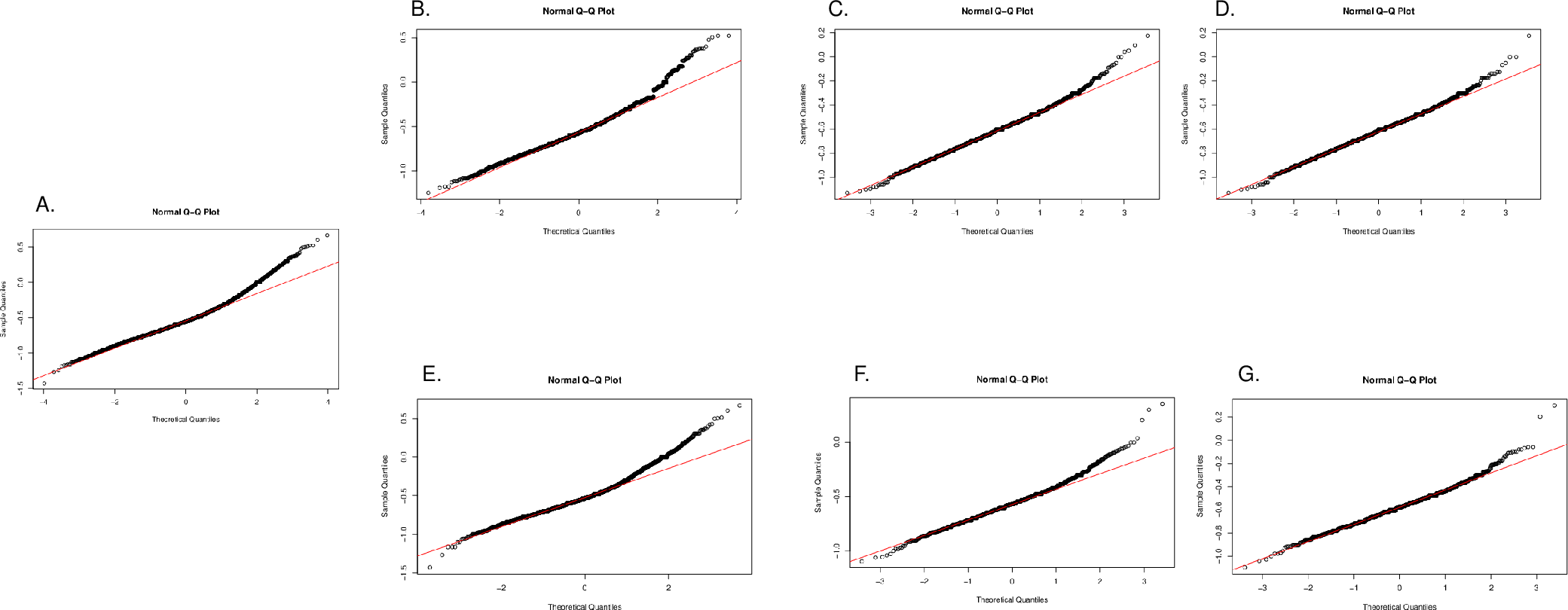

Supplement: S2 Fig — A. All anonymized samples: females n = 9972; males n = 4583. From left to right, QQ plots are separated by sex with females on the top line (B, C, D) and males beneath (E, F, G). B and E are all anonymized samples; C (n = 2661) and F (n = 1593) are those with normal hematological indices, but without any limits for monocytes and lymphocytes; D and G are those with normal hematological indices, but with upper limits of 0.8 x 10^9^/L for monocytes and 4 x 10^9^/L for lymphocytes in females (n = 2550) and males (n = 1426) respectively (TB clinic CBCs not removed). (TIF) [file pone.0247745.s002.tif]

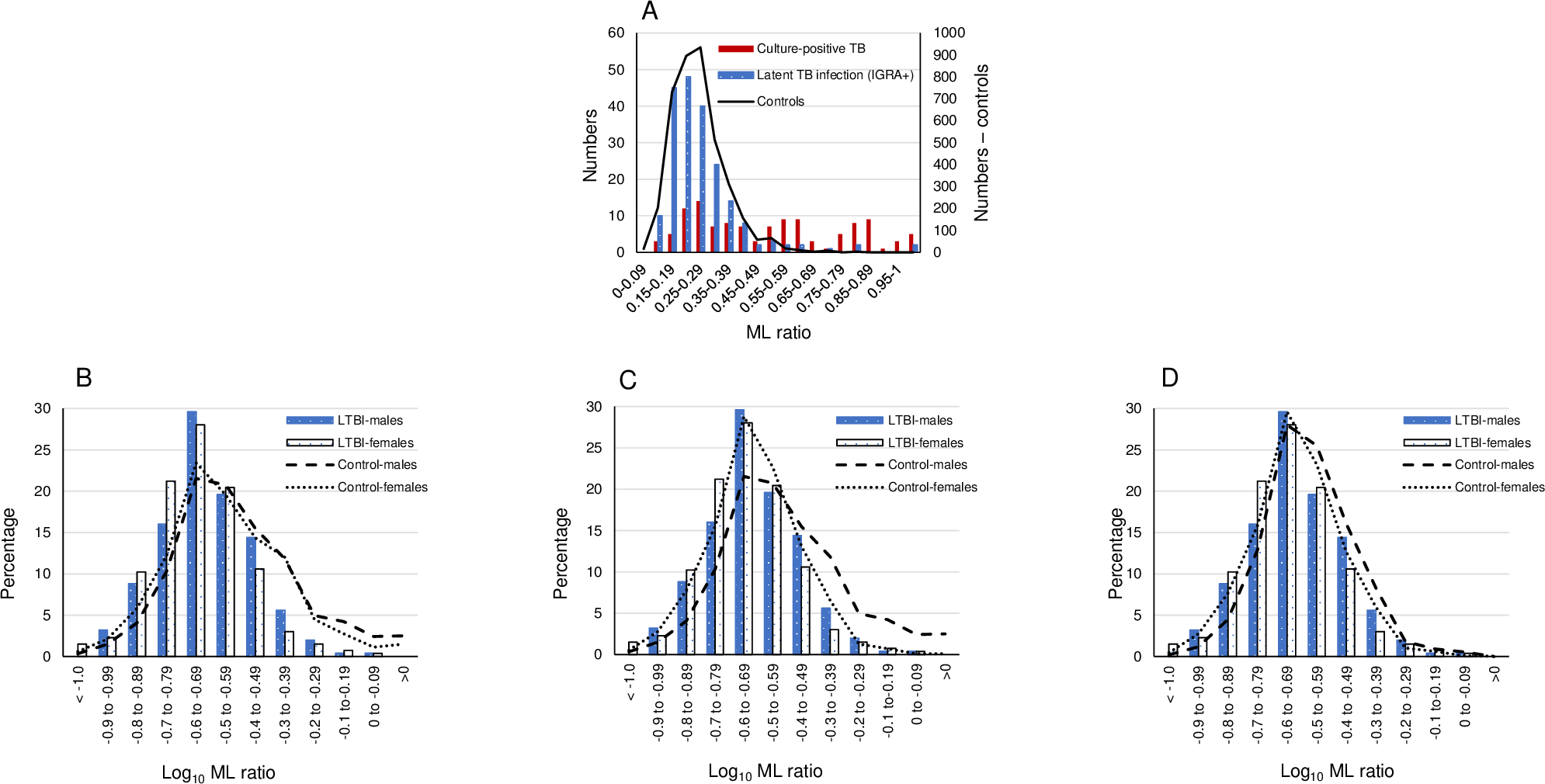

Supplement: S3 Fig — A. MLRs comparing culture-positive TB, LTBI and hospital CBCs with normal hematological indices. B. Log. transformed MLRs: all CBCs. C. Log. transformed MLRs: CBCs with normal hematological indices but no restriction on monocyte or lymphocyte limits. D. Log. transformed MLRs: CBCs with normal hematological indices, monocytes < 0.8 x 10^9^/L and lymphocyte < 4.0 x 10^9^/L. (TIF) [file pone.0247745.s003.tif]
