# Supplementary material for: The monocyte-to-lymphocyte ratio: Sex-specific differences in the tuberculosis disease spectrum, diagnostic indices and defining normal ranges
Source: PLoS One. 2021 Aug 30;16(8):e0247745. doi: 10.1371/journal.pone.0247745 (PMC8405018; doi:10.1371/journal.pone.0247745)
Supplement: S1 File — (DOCX) [file pone.0247745.s004.docx]

**Online Data Supplement**

**The monocyte-to-lymphocyte ratio: defining a normal range, sex-specific differences in the tuberculosis disease spectrum and diagnostic indices**

**Multivariate analyses of patient predictors and MLR**

Thomas S. Buttle^1¶#a^, Claire Y. Hummerstone^1&^, Thippeswamy Billahalli^1&^, Richard J. B. Ward^1&^, Korina E. Barnes^2#b^, Natalie J. Marshall^2^, Viktoria C. Spong^1&^, Graham H. Bothamley^1,3,4¶*^

^1^ Department of Respiratory Medicine, Homerton University Hospital, London E9 6SR, UK

^2^ Microbiology Department, Homerton University Hospital, London E9 6SR, UK

^3^ Department of Immunobiology, Blizard Institute, Barts and The London School of Medicine and Dentistry, Queen Mary University of London, London E1 2AD, UK

^4^ Department of Infectious and Tropical Diseases, London School of Hygiene and Tropical Medicine, London WC1E 7HT, UK

^#a^ Department of Respiratory Medicine, Princess Royal University Hospital, King’s College NHS Foundation Trust, Farnborough Common, Orpington, BR6 8ND, UK

^#b^ Department of Microbiology, Great Western Hospitals NHS Trust, Marlborough Road, Swindon, SN3 6BB, UK.

* Corresponding author

E-mail: [g.bothamley@nhs.net](mailto:g.bothamley@nhs.net)

^¶^These authors contributed equally to this work

^&^These authors contributed equally to this work

**Multiple linear regression analysis of TB population cohort**

**Model 1a**

Factors examined site of disease (pulmonary vs extrapulmonary), smear, culture, white blood count, albumin, globulin, AG ratio and Log CRP. However, there were many values < 5 mg/L and several missing values for Log CRP, so that this predictor was removed for the analysis of the other factors.

| **Predictor** | **Coefficient**  **estimate** | **Standard Error** | **t-statistic** | **p-value** |
| --- | --- | --- | --- | --- |
| Constant | 0.582 | 0.73 | 0.797 | 0.426 |
| Albumin | 0.092 | 0.133 | 0.691 | 0.49 |
| White cell count | 0.013 | 0.005 | 2.563 | 0.011 |
| Culture | 0.042 | 0.038 | 1.11 | 0.268 |
| AG ratio | -0.018 | 0.493 | -0.591 | 0.555 |
| Smear | -0.076 | 0.129 | -1.743 | 0.08 |
| Pulmonary vs extrapulmonary | -0.055 | 0.059 | -0.947 | 0.345 |
| Globulin | -0.011 | 0.014 | -0.832 | 0.407 |

None of the interactions Alb*AG, AG*Globulin, Smear*P vs EP, Culture*P vs EP nor Smear*Culture was significant.

R-squared = 0.34, adjusted R-squared = 0.304

F-statistic = 9.347, p = 0

Histogram of residuals 53:47

Normal probability plot of residuals was a straight line (homoscedasticity assumption met)

**Model 1b**

Best fit model without CRP

| **Predictor** | **Coefficient**  **estimate** | **Standard Error** | **t-statistic** | **p-value** |
| --- | --- | --- | --- | --- |
| Constant | -0.0523 | 0.0971 | -0.5381 | 0.591 |
| Albumin | -0.0151 | 0.0021 | -7.1978 | 0 |
| WBC | 0.0157 | 0.0048 | 3.2482 | 0.0013 |
| Culture | 0.0948 | 0.0287 | 3.3053 | 0.0011 |

R-squared = 0.313, adjusted R-squared = 0.3048

Analysis of variance

| **Source** | **df** | **SS** | **MS** | **F-statistic** | **P-value** |
| --- | --- | --- | --- | --- | --- |
| Regression | 3 | 5.2736 | 1.7579 | 37.9666 | 0 |
| Residual error | 250 | 11.575 | 0.0463 |  |  |
| Total | 253 | 16.8486 | 0.0666 |  |  |

Histogram of residuals 49.5:50.5.

Normal probability of residuals above the line in the 4^th^ quartile (min = -0.6001, 1^st^ quartile = -0.1336, median = 0.0019, 3^rd^ quartile = 0.1267, max = 1.0165).

**Model 2**

Males

Log CRP showed a significant association with albumin levels and was excluded.

The three main predictors gave a model which was significant but the coefficient for the wbc had fallen to give P = 0.061.

**Model 2a. Using model 1 predictors**

| **Predictor** | **Coefficient**  **estimate** | **Standard Error** | **t-statistic** | **P-value** |
| --- | --- | --- | --- | --- |
| Constant | -0.007 | 0.116 | -0.058 | 0.954 |
| Albumin | -0.015 | 0.002 | -6.376 | 0 |
| Culture | 0.12 | 0.036 | 3.332 | 0.001 |
| White cell count | 0.012 | 0.006 | 1.887 | 0.061 |

R-squared = 0.340, adjusted R-squared = 0.328

Analysis of variance

| **Source** | **df** | **SS** | **MS** | **F-statistic** | **P-value** |
| --- | --- | --- | --- | --- | --- |
| Regression | 3 | 4.072 | 1.357 | 28.003 | 0 |
| Residual error | 163 | 7.902 | 0.048 |  |  |
| Total | 166 | 11.974 | 0.072 |  |  |

Histogram of residuals 50:40

Normal probability of residuals linear (min = -0.621, 1^st^ quartile = -0.136, median = 0.001, 3^rd^ quartile = 0.122, max = 0.991)

If Log CRP were added, only Log CRP and HIV gave significant P-values for coefficients using culture and only Log CRP using smear.

Removing the white cell count as a predictor, then required the addition of AG ratio (with no interaction with albumin) and HIV, but the new model gave better adjusted R-squared values and smaller sum of the squares.

**Model 2b**

| **Predictor** | **Coefficient**  **estimate** | **Standard Error** | **t-statistic** | **P-value** |
| --- | --- | --- | --- | --- |
| Constant | 0.24 | 0.092 | 2.61 | 0.01 |
| Albumin | -0.025 | 0.004 | -5.871 | 0 |
| Culture | 0.129 | 0.036 | 3.566 | 0 |
| AG ratio | 0.187 | 0.105 | 1.788 | 0.076 |
| HIV | -0.385 | 0.109 | -3.541 | 0.001 |

R-squared = 0.411, adjusted R-squared = 0.395

Analysis of variance

| **Source** | **df** | **SS** | **MS** | **F-statistic** | **P-value** |
| --- | --- | --- | --- | --- | --- |
| Regression | 4 | 4.744 | 1.186 | 26.478 | 0 |
| Residual error | 152 | 6.809 | 0.045 |  |  |
| Total | 156 | 11.553 | 0.074 |  |  |

Histogram of residuals 51:49

Normal probability of residuals linear (min = -0.586, 1^st^ quartile = -0.141, median = -0.004, 3^rd^ quartile = 0.123, max = 0.95)

**Model 3**

Females

Using model 1 predictors

| **Predictor** | **Coefficient**  **estimate** | **Standard Error** | **t-statistic** | **p-value** |
| --- | --- | --- | --- | --- |
| Constant | 0.442 | 0.297 | 1.488 | 0.141 |
| White cell count | 0.023 | 0.008 | 3.044 | 0.003 |
| Albumin | -0.03 | 0.008 | -3.885 | 0 |
| Culture | -0.699 | 0.333 | -2.1 | 0.039 |
| Culture*albumin | 0.021 | 0.009 | 2.264 | 0.026 |

R-squared = 0.319, adjusted R-squared = 0.285

Analysis of variance

| **Source** | **df** | **SS** | **MS** | **F-statistic** | **P-value** |
| --- | --- | --- | --- | --- | --- |
| Regression | 4 | 1.509 | 0.377 | 9.583 | 0 |
| Residual error | 82 | 3.228 | 0.039 |  |  |
| Total | 86 | 4.737 | 0.055 |  |  |

Histogram 52:48.

Normal probability of residuals above the line in the 4^th^ quartile (min = -0.491, 1^st^ quartile = -0.134, median = -0.013, 3^rd^ quartile = 0.133, max = 0.475).

If Log CRP were added, it became the only variable with a significant coefficient. No other model could improve on the model 1 predictors.

**Multilinear regression analysis of S+PTB**

**Model 1 – with Log CRP**

Variables examined were Log CRP, albumin-to-globulin ratio, albumin, neutrophil count, sex globulin, cavities and smear.

Removing non-significant predictors one at a time, the final model where each coefficient was significant was

Log ML = 0.652 -0.409 Log CRP + 0.11 sex – 0.038 albumin + 0.013 neutrophils + 0.017 Log CRP * alb

| **Predictor** | **Coefficient**  **estimate** | **Standard Error** | **t-statistic** | **p-value** |
| --- | --- | --- | --- | --- |
| Constant | 0.652 | 0.351 | 1.854 | 0.065 |
| Log CRP | -0.409 | 0.175 | -2.346 | 0.02 |
| Sex | 0.11 | 0.031 | 3.544 | 0 |
| Albumin | -0.038 | 0.01 | -3.93 | 0 |
| Neutrophil count | 0.013 | 0.005 | 2.598 | 0.01 |
| LogCRP * albumin | 0.017 | 0.005 | 3.317 | 0.001 |

R-squared = 0.252, adjusted R-squared = 0.236

Analysis of variance

| **Source** | **df** | **SS** | **MS** | **F-statistic** | **P-value** |
| --- | --- | --- | --- | --- | --- |
| Regression | 5 | 4.118 | 0.824 | 16.142 | 0 |
| Residual error | 240 | 12.245 | 0.051 |  |  |
| Total | 245 | 16.362 | 0.067 |  |  |

Histogram 51:49.

Normal probability of residuals above the line in the 4^th^ quartile (min = -0.743, 1^st^ quartile = -0.147, median = -0.011, 3^rd^ quartile = 0.123, max = 0.967).

**Model 2 – without Log CRP**

There were many missing values for CRP, so the modelling was repeated without CRP, again beginning with all predictors that had a value P < 0.1 in the univariate analysis.

| **Predictor** | **Coefficient**  **estimate** | **Standard Error** | **t-statistic** | **p-value** |
| --- | --- | --- | --- | --- |
| Constant | -0.034 | 0.075 | -0.458 | 0.647 |
| Sex | 0.1 | 0.029 | 3.429 | 0.001 |
| Albumin | -0.12 | 0.002 | -6.05 | 0 |
| Neutrophil count | 0.017 | 0.005 | 3.472 | 0.001 |

R-squared = 0.1895, adjusted R-squared = 0.1808

Analysis of variance

| **Source** | **df** | **SS** | **MS** | **F-statistic** | **P-value** |
| --- | --- | --- | --- | --- | --- |
| Regression | 3 | 3.843 | 1.281 | 23.087 | 0 |
| Residual error | 284 | 15.759 | 0.055 |  |  |
| Total | 287 | 19.602 | 0.068 |  |  |

Histogram 50.5:49.5

Normal probability of residuals above the line in the 4^th^ quartile (min = -0.877, 1^st^ quartile = -0.147, median = -0.001, 3^rd^ quartile = 0.129, max = 0.959).

**Multilinear regression by sex for S+PTB**

**Model 1 (Females)**

Use Log neutrophil count, Log CRP, AG ratio. albumin, smear, HIV, drug-resistance, globulin, cavities, EtOH, (all P<0.1 in univariate analysis)

| **Predictor** | **Coefficient**  **estimate** | **Standard Error** | **t-statistic** | **p-value** |
| --- | --- | --- | --- | --- |
| Constant | -0.059 | 1.639 | -0.036 | 0.972 |
| Albumin | -0.106 | 0.436 | -0.242 | 0.81 |
| Log neutrophil count | 0.318 | 0.137 | 2.322 | 0.025 |
| Log CRP | 0.316 | 0.509 | 0.62 | 0.539 |
| AG ratio | -1.827 | 1.272 | -1.437 | 0.158 |
| Smear grade | 0.049 | 0.051 | 0.952 | 0.346 |
| HIV | -0.09 | 0.123 | -0.734 | 0.467 |
| globulin | -0.02 | 0.013 | -1.566 | 0.125 |
| Cavities | 0.256 | 0.163 | 1.574 | 0.123 |
| EtOH | 0.005 | 0.284 | 0.019 | 0.985 |
| Drug resistance | -0.15 | 0.135 | -1.112 | 0.272 |

No interaction was significant

R-squared = 0.502, adjusted R-squared = 0.317

Analysis of variance

| **Source** | **df** | **SS** | **MS** | **F-statistic** | **P-value** |
| --- | --- | --- | --- | --- | --- |
| Regression | 16 | 2.236 | 0.14 | 2.712 | 0.005 |
| Residual error | 43 | 2.215 | 0.052 |  |  |
| Total | 59 | 4.451 | 0.075 |  |  |

Histogram 47:53.

Normal probability of residuals above the line in the 4^th^ quartile (min = -0.456, 1^st^ quartile = -0.141, median = 0.02, 3^rd^ quartile = 0.139, max = 0.453).

[Only albumin showed a significant association]

**Model 1b**

| **Predictor** | **Coefficient**  **estimate** | **Standard Error** | **t-statistic** | **p-value** |
| --- | --- | --- | --- | --- |
| Constant | -1.229 | 0.171 | -7.192 | 0 |
| Log neutrophil count | 0.276 | 0.125 | 2.204 | 0.032 |
| Log CRP | 0.277 | 0.078 | 3.532 | 0.001 |
| Smear grade | 0.07 | 0.029 | 2.393 | 0.02 |
| Cavities | 0.121 | 0.059 | 2.066 | 0.044 |

R-squared = 0.39, adjusted R-squared = 0.346

Analysis of variance

| **Source** | **df** | **SS** | **MS** | **F-statistic** | **P-value** |
| --- | --- | --- | --- | --- | --- |
| Regression | 4 | 1.738 | 0.434 | 8.804 | 0 |
| Residual error | 55 | 2.714 | 0.049 |  |  |
| Total | 59 | 4.451 | 0.075 |  |  |

Histogram 53:47.

Normal probability of residuals above the line in the 4^th^ quartile (min = -0.68, 1^st^ quartile = -0.119, median = -0.015, 3^rd^ quartile = 0.117, max = 0.355).

**Model 1c**

| **Predictor** | **Coefficient**  **estimate** | **Standard Error** | **t-statistic** | **p-value** |
| --- | --- | --- | --- | --- |
| Constant | -0.355 | 0.112 | -3.16 | 0.002 |
| Log neutrophil count | 0.365 | 0.105 | 3.493 | 0.001 |
| AG | -0.381 | 0.093 | -4.085 | 0 |
| Cavities | 0.14 | 0.047 | 2.977 | 0.004 |

R-squared = 0.35, adjusted R-squared = 0.328

Analysis of variance

| **Source** | **df** | **SS** | **MS** | **F-statistic** | **P-value** |
| --- | --- | --- | --- | --- | --- |
| Regression | 3 | 2.226 | 0.742 | 15.455 | 0 |
| Residual error | 86 | 4.13 | 0.048 |  |  |
| Total | 89 | 6.356 | 0.071 |  |  |

Histogram 47:53.

Normal probability of residuals above the line in the 4^th^ quartile (min = -0.725, 1^st^ quartile = -0.126, median = -0.022, 3^rd^ quartile = 0.156, max = 0.545).

**Model 1d**

| **Predictor** | **Coefficient**  **estimate** | **Standard Error** | **t-statistic** | **p-value** |
| --- | --- | --- | --- | --- |
| Constant | -0.534 | 0.134 | -3.985 | 0 |
| Log neutrophil count | 0.33 | 0.102 | 3.232 | 0.002 |
| AG | -0.344 | 0.095 | -3.612 | 0.001 |
| Smear | 0.082 | 0.033 | 2.462 | 0.016 |
| Cavities | 0.368 | 0.109 | 3.364 | 0.001 |
| Smear*cavities | -0.104 | 0.046 | -2.26 | 0.026 |

R-squared = 0.407, adjusted R-squared = 0.372

Analysis of variance

| **Source** | **df** | **SS** | **MS** | **F-statistic** | **P-value** |
| --- | --- | --- | --- | --- | --- |
| Regression | 5 | 2.567 | 0.513 | 11.404 | 0 |
| Residual error | 83 | 3.737 | 0.045 |  |  |
| Total | 88 | 6.304 | 0.072 |  |  |

Histogram 47:53.

Normal probability of residuals above the line in the 4^th^ quartile (min = -0.725, 1^st^ quartile = -0.126, median = -0.022, 3^rd^ quartile = 0.156, max = 0.545).

**Model (Males)**

Use Log CRP, AG ratio, albumin, globulin, neutrophils

The coefficient for neutrophils and albumin-to-globulin ratio were non-significant (P=0.3891and 0.6687 respectively)

Repeating without the AG ratio, the coefficient for neutrophils remained non-significant (P=0.3858)

| **Predictor** | **Coefficient**  **estimate** | **Standard Error** | **t-statistic** | **p-value** |
| --- | --- | --- | --- | --- |
| Constant | 1.020 | 0.399 | 2.559 | 0.011 |
| Log CRP | -0.495 | 0.199 | -2.487 | 0.014 |
| Albumin | -0.042 | 0.011 | -43.806 | 0 |
| Log CRP*albumin | 0.018 | 0.006 | 3.172 | 0.002 |

R-squared = 0.197, adjusted R-squared = 0.182

Analysis of variance

| **Source** | **df** | **SS** | **MS** | **F-statistic** | **P-value** |
| --- | --- | --- | --- | --- | --- |
| Regression | 3 | 1.903 | 0.634 | 13.264 | 0 |
| Residual error | 162 | 7.748 | 0.048 |  |  |
| Total | 165 | 9.651 | 0.058 |  |  |

Histogram 52:48.

Normal probability of residuals above the line in the 4^th^ quartile (min = -0.595, 1^st^ quartile = -0.148, median = -0.009, 3^rd^ quartile = 0.12, max = 0.98).
