# Supplementary material for: The monocyte-to-lymphocyte ratio: Sex-specific differences in the tuberculosis disease spectrum, diagnostic indices and defining normal ranges
Source: PLoS One. 2021 Aug 30;16(8):e0247745. doi: 10.1371/journal.pone.0247745 (PMC8405018; doi:10.1371/journal.pone.0247745)
Supplement: S2 File — (DOCX) [file pone.0247745.s005.docx]

**Online Data Supplement**

**The monocyte-to-lymphocyte ratio: sex-specific differences in the tuberculosis disease spectrum, diagnostic indices and defining normal ranges**

**Supplementary Tables and Legends to Supplementary Figures**

Thomas S. Buttle^1¶#a^, Claire Y. Hummerstone^1&^, Thippeswamy Billahalli^1&^, Richard J. B. Ward^1&^, Korina E. Barnes^2#b^, Natalie J. Marshall^2^, Viktoria C. Spong^1&^, Graham H. Bothamley^1,3,4¶*^

^1^ Department of Respiratory Medicine, Homerton University Hospital, London E9 6SR, UK

^2^ Microbiology Department, Homerton University Hospital, London E9 6SR, UK

^3^ Department of Immunobiology, Blizard Institute, Barts and The London School of Medicine and Dentistry, Queen Mary University of London, London E1 2AD, UK

^4^ Department of Infectious and Tropical Diseases, London School of Hygiene and Tropical Medicine, London WC1E 7HT, UK

^#a^ Department of Respiratory Medicine, Princess Royal University Hospital, King’s College NHS Foundation Trust, Farnborough Common, Orpington, BR6 8ND, UK

^#b^ Department of Microbiology, Great Western Hospitals NHS Trust, Marlborough Road, Swindon, SN3 6BB, UK.

* Corresponding author

E-mail: [g.bothamley@nhs.net](mailto:g.bothamley@nhs.net)

^¶^These authors contributed equally to this work

^&^These authors contributed equally to this work

**S1 Table**. **Demographic data on patients with sputum smear-positive pulmonary tuberculosis**

|  | 2014-17  (n=61) | 2005-13  (n=236) | Total  (n=297) |
| --- | --- | --- | --- |
| **Age**:  median (range), years | 41 (18-87) | 38 (17-87) | 36 (17-87) |
| **Female sex**: number (%) | 19 (32) | 82 (35) | 101 (34) |
| **Ethnicity**: number (%)  Indian subcontinent  Black African  Afro-Caribbean  Turkish/Kurdish  White EU  White UK  Mixed  Other | 8 (13)  9 (15)  13 (21)  6 (10)  8 (13)  7 (12)  3 (5)  7 (12) | 33 (14)  65 (28)  39 (17)  12 (5)  17 (7)  34 (15)  8 (3)  28 (12) | 41 (14)  74 (25)  52 (18)  18 (6)  25 (9)  41 (14)  11 (4)  35 (12) |
| **UK-born**: number (%) | 18 (30) | 78 (33) | 96 (32) |
| **Previous TB**: number (%) | 8 (14)* | 26 (11) | 34 (12) |
| **IGRA**: number (%)*  Positive  Negative  Indeterminate  Not done | 16 (89)*  2 (11)*  0 (0)*  43 (72) | 50 (82)*  6 (10)*  5 (8)*  175 (74) | 66 (84)*  8 (10)*  5 (6)*  218 (74) |
| **BCG**: number (%) | 42 (78)* | 166 (73)* | 208 (74)* |
| **HIV coinfection**: number (%) | 3 (5)* | 17 (8)* | 20 (7)* |
| **Diabetes**: number (%) | 10 (17) | 21 (9) | 31 (10) |
| **Alcohol problem:** number (%) | 16 (27) | 55 (23) | 71 (24) |
| **Family or direct contact;** number (%) | 6 (10) | 60 (26) | 66 (22) |
| **Sputum score:** number (%)  +/-  +  ++  +++ | 1 (4)  10 (18)  13 (23)  33 (58) | 28 (12)  35 (15)  38 (17)  127 (56) | 29 (10)  45 (16)  51 (18)  160 (56) |
| **Drug-resistance:** number (%)  Isoniazid  Rifampicin  Pyrazinamide  MDR-TB | 12 (20)  1 (2)  0 (0)  1 (2) | 19 (29)  0 (0)  0(0)  0(0) | 31 (25)  1 (1)  0 (0)  1 (1) |
| **Chest radiographs**  Zones: number (%)  Normal  1-2  3-4  >4  Cavitation present: number (%) | 3 (5)  36 (59)  17 (28)  5 (8)  32 (53) | 3 (1)  138 (59)  74 (31)  21 (9)  46 (62) | 6 (2)  174 (59)  91 (31)  26 (9)  177 (60) |
| **White blood count > 11 x 10^9^/L**: number (%) | 14 (24) | 38 (15) | 52 (17) |
| **Albumin < 40 g/L:** number (%) | 54 (94) | 204 (90)* | 260 (88)* |
| **Globulin >32 g/L:** number (%) | 57 (96)* | 219 (94)* | 276 (95*) |
| **CRP > 10 mg/L:** number (%) | 50 (86)* | 191 (85)* | 241 (86)* |

*indicates percentage of those tested or examined or with available data

**S2 Table**. **Normal ranges for full blood counts (SI units).**

| **Variable** | **Male** | **Female** |
| --- | --- | --- |
| Hemoglobin (g/L) | 13 - 18 | 11.5 – 16.5 |
| Red blood cell count (x 10^9^/L) | 4.5 – 6.5 | 3.8 – 5.8 |
| Packed cell volume | 0.4 – 0.54 | 0.37 – 0.47 |
| Mean cell volume (fL) | 80 – 98 | |
| Mean cell hemoglobin (pg/cell) | 27 – 32 | |
| RDW – red cell distribution width (%) | 10 – 14 | |
| Platelets (x 10^9^/L) | 150 – 600 | |
| White blood cell count (x 10^9^/L) | 4.0 – 11.0 | |
| Neutrophils (x 10^9^/L) | 2.0 – 7.5 | |
| Lymphocytes (x 10^9^/L) | 1.0 – 4.0 | |
| Monocytes (x 10^9^/L) | 0.2 – 1.0^a^ | |
| Eosinophils (x 10^9^/L) | 0 – 0.4 | |
| Basophils (x 10^9^/L) | 0 – 0.1 | |

^a^ An upper limit of 0.8 x 10^9^/L was used following Hensel M et al. Peripheral monocytosis as a predictive factor for adverse outcome in the emergency department. Medicine 2017; 96:28(e7404).

**S3 Table. Diagnostic indices using ML cut-offs of 0.515/0.118 for females and 0.595/0.129 for males.**

| Population | Sex | No. | True positives | False positives | False negatives | True negatives | Sensitivity  (%) | Specificity  (%) |
| --- | --- | --- | --- | --- | --- | --- | --- | --- |
| **General CBCs** ^a^ | F | 9977 | 0 (0)^b^ | 1219 (1029) | 2 (2) | 8756 (8946) | 0 (0) | 88 (90) |
|  | M | 4594 | 1 (1) | 623  (536) | 8 (8) | 3762 (4049) | 22 (22) | 82 (88) |
| **TB notifications**^c^ | F | 112 | 26 (25) | 5 (3) | 62 (63) | 19 (21) | 30 (29) | 79 (88) |
|  | M | 203 | 57 (52) | 4 (4) | 119 (124) | 23 (23) | 32 (30) | 85 (85) |
| **All screened excluding contacts** | F | 251 | 5 (4) | 11 (5) | 51 (52) | 184 (190) | 8.9 (7.1) | 94 (98) |
|  | M | 233 | 11 (9) | 11 (3) | 45 (47) | 166 (174) | 20 (16) | 92 (98) |
| **Contacts of S+PTB index vs. S+PTB diagnosed after screening** | F | 106 | 1 (1) | 3 (2) | 35 (35) | 66 (67) | 2.7 (2.7) | 96 (97) |
|  | M | 117 | 0 (0) | 3 (1) | 44 (35) | 73 (75) | 0 (0) | 96 (99) |

CBC=complete blood count; F=female; M=male; TB = tuberculosis; S+PTB=sputum smear-positive pulmonary tuberculosis. ^a^ Sex for two samples not available. ^b^ Figures in brackets are for upper limits only. ^c^ 50 denotified cases included.

**S4 Table**. **Sensitivity and specificity of sex-specific MLR cut-off values^a^**

| Population | Sex | No. | True positives | False positives | False negatives | True negatives | Sensitivity  (%) | Specificity  (%) |
| --- | --- | --- | --- | --- | --- | --- | --- | --- |
| **General CBCs** ^b^ | F | 9977 | 0 (0)^c^ | 1552 (1297) | 2 (2) | 8423 (8678) | 0 (0) | 85 (87) |
|  | M | 4594 | 2 (2) | 794 (686) | 7 (7) | 3791 (3899) | 22 (22) | 83 (85) |
| **TB notifications**^d^ | F | 112 | 28 (27) | 7 (5) | 60 (61) | 17 (19) | 32 (31) | 71 (79) |
|  | M | 203 | 74 (69) | 8 (7) | 102 (107) | 19 (20) | 42 (39) | 70 (74) |
| **All screened excluding contacts** | F | 251 | 8 (7) | 11 (5) | 48 (49) | 184 (190) | 14 (13) | 94 (98) |
|  | M | 233 | 14 (11) | 14 (4) | 42 (45) | 163 (173) | 25 (20) | 92 (98) |
| **Contacts of S+PTB index vs. S+PTB diagnosed after screening** | F | 106 | 6 (5) | 3 (2) | 30 (31) | 67 (68) | 17 (14) | 96 (97) |
|  | M | 117 | 11 (9) | 8 (3) | 33 (35) | 65 (70) | 25 (21) | 89 (96) |

^a^ Mean ± 2SD of general CBCs without any hematological abnormality and with monocytes < 0.8 x 10^9^/L, i.e. females < 0.122 and > 0.474, males < 0.136 and > 0.505.

CBC=complete blood count; F=female; M=male; TB = tuberculosis; S+PTB=sputum smear-positive pulmonary tuberculosis. ^b^ Sex for two samples not available. ^c^ Figures in brackets are for upper limits only. ^d^ 50 denotified cases included.

**S5 Table**. **Sensitivity and specificity and the ML ratio in total CBCs and TB notifications**

| Population: criteria^a^ | No.^b^ | True positives | False positives^c^ | False negatives | True negatives | Sensitivity  (%) | Specificity  (%) |
| --- | --- | --- | --- | --- | --- | --- | --- |
| **1 month**  <0.17  >0.20  Combined  0.17-0.20  <0.09  >0.25  Combined  0.09-0.25 | **14,573**  1,560  11,288  12,838  1,735  49  8,461  8,483  6,079 | 0  9  9  0  6  6 | 1,560  11,279  12,839  49  8,455  8,504 | 1  4 | 14,503  6,076 | 90  60 | 100  100 |
| **TB notifications**  <0.17  >0.20  Combined  0.17-0.20  <0.09  >0.25  Combined  0.09-0.25 | **314^b^**  20  274  294  20  0  242  242  72 | 15  234  249  0  207  207 | 5  40  45  0  35  35 | 15  57 | 5  15 | 94  78 | 10  30 |

^a^ Criteria derived from Wang J et al. Braz J Infect Dis 2015; 19: 125-31, either using 9^th^ and 25^th^ centiles dereived from our population (<0.17 and >0.20) or actual values (<0.09 and >0.25) from their paper

^b^ Includes only those with a full blood count with data permitting calculation of the ML ratio

^c^ False positives and negatives include denotified group, where a firm alternative diagnosis was reached after having previously been notified as a case of tuberculosis.

**S6 Table. Value of other published MLR cut-offs in contacts screened for TB**

| Cut-offs defined by other publications | No. | True positives^a^ | False positives^b^ | False negatives^a^ | True negatives^b^ | Sensitivity  (%) | Specificity  (%) |
| --- | --- | --- | --- | --- | --- | --- | --- |
| **Wang et al. (I) ^c^**  <0.17  >0.20  Combined  0.17-0.20  **Wang et al. (II)^d^**  <0.09  >0.25  Combined^d^  0.09-0.25  **ERJ 2015**^e^  TST ≥ 14 mm MC > 0.8x10^9^/L  Combined | 11  84  95  10  0  76  76  29  134  134  134 | 11  84  95  0  76  76  47  6  48 | 13  72  85  0  48  48  61  2  62 | 10  29  6  47  5 | 15  52  20  79  19 | 91  72  89  11  91 | 15  52  25  98  24 |

^a^ Diagnosed with TB at screening

^b^ Positive IGRA but no TB at screening

^c^ After Wang J et al. Braz J Infect Dis 2015; 19: 125-31, using ML ratios of 9^th^ and 25^th^ centiles found in our general population:

^d^ Actual MLRs used by Wang J et al. Braz J Infect Dis 2015; 19: 125-31

^e^ Rakotosamimanana N et al. Eur Resp J 2015; 46: 1095-1103. Few of our patients with TB had a tuberculin skin test (TST)

**Legend to supplementary Figures.**

**S1 Fig. Monocyte-to-lymphocyte ratio (MLR) and neutrophil count.**

**S2 Fig. QQ plots for log. ML ratios for unselected and those with normal hematological indices.**

**A.** All anonymized samples: females n=9972; males n=4583. From left to right, QQ plots are separated by sex with females on the top line (**B**, **C**, **D**) and males beneath (**E**, **F**, **G**). **B** and **E** are all anonymized samples; **C** (n=2661) and **F** (n=1593) are those with normal hematological indices, but without any limits for monocytes and lymphocytes; **D** and **G** are those with normal hematological indices, but with upper limits of 0.8 x 10^9^/L for monocytes and 4 x 10^9^/L for lymphocytes in females (n=2550) and males (n=1426) respectively (TB clinic CBCs not removed).

**S3 Fig.** **Comparison between screened population and CBCs:** **effect of choosing different limits for the monocyte and lymphocyte counts**

**A**. MLRs comparing culture-positive TB, LTBI and hospital CBCs with normal hematological indices. **B**. Log. transformed MLRs: all CBCs. **C**. Log. transformed MLRs: CBCs with normal hematological indices but no restriction on monocyte or lymphocyte limits. **D**. Log. transformed MLRs: CBCs with normal hematological indices, monocytes < 0.8 x 10^9^/L and lymphocyte < 4.0 x 10^9^/L.
